# Intake of Soy, Soy Isoflavones and Soy Protein and Risk of Cancer Incidence and Mortality

**DOI:** 10.3389/fnut.2022.847421

**Published:** 2022-03-04

**Authors:** Yahui Fan, Mingxu Wang, Zhaofang Li, Hong Jiang, Jia Shi, Xin Shi, Sijiao Liu, Jinping Zhao, Liyun Kong, Wei Zhang, Le Ma

**Affiliations:** ^1^The First Affiliated Hospital, Xi'an Jiaotong University Health Science Center, Xi'an, China; ^2^Key Laboratory of Shaanxi Province for Craniofacial Precision Medicine Research, College of Stomatology, Xi'an Jiaotong University, Xi'an, China; ^3^School of Public Health, Xi'an Jiaotong University Health Science Center, Xi'an, China; ^4^Key Laboratory for Disease Prevention and Control and Health Promotion of Shaanxi Province, Xi'an, China; ^5^Key Laboratory of Environment and Genes Related to Diseases (Xi'an Jiaotong University), Ministry of Education of China, Xi'an, China

**Keywords:** soy, soy isoflavones, soy protein, cancer, meta-analysis

## Abstract

**Background and Aims:**

Associations between soy intake and risk of cancer have been evaluated in prospective observational studies with inconsistent results. Whether the potential anticancer effects offered by soy were attributed to soy isoflavones and soy protein still needs to be elucidated. This study aimed to comprehensively quantify the association of soy, soy isoflavones and soy protein intake with risk of cancer incidence and cancer mortality by conducting a meta-analysis of all available studies.

**Methods:**

PubMed, Embase, Web of Science, and Cochrane Library databases were searched up to 16 September 2021. Prospective cohort studies that examined the effect of soy, soy isoflavones and soy protein on cancer incidence and cancer mortality were identified. Random-effects models were used to pool the multivariable-adjusted relative risks (RRs) and corresponding 95% confidence intervals (CIs). The potential dose-response relations were explored by using generalized least-squares trend estimation.

**Results:**

Eighty one prospective cohort studies were included in the meta-analysis. A higher intake of soy was significantly associated with a 10% reduced risk of cancer incidence (RR, 0.90; 95% CI, 0.83–0.96). Each additional 25 g/d soy intake decreased the risk of cancer incidence by 4%. Intake of soy isoflavones was inversely associated with risk of cancer incidence (RR, 0.94; 95% CI, 0.89–0.99), whereas no significant association was observed for soy protein. The risk of cancer incidence was reduced by 4% with each 10 mg/d increment of soy isoflavones intake. Similar inverse associations were also found for soy in relation to site-specific cancers, particularly lung cancer (RR, 0.67; 95%CI, 0.52–0.86) and prostate cancer (RR, 0.88; 95%CI, 0.78–0.99). However, high intake of soy, soy isoflavones and soy protein were not associated with cancer mortality.

**Conclusions:**

Higher intake of soy and soy isoflavones were inversely associated with risk of cancer incidence, which suggested that the beneficial role of soy against cancer might be primarily attributed to soy isoflavones. These findings support recommendations to include soy as part of a healthy dietary pattern for the prevention of cancer.

## Introduction

Soybeans are considered as key components of plant-based dietary patterns and dietary guidelines from several organizations recommend their increasing consumption for the prevention of heart disease and other chronic conditions (1, 2). Such health benefits may be attributed to the multiple nutrients and associated phytochemicals of soy (3). As the major nutritional profile of soy, soy isoflavones and soy protein have been suggested to reduce oxidative stress and inflammation (4, 5), both of which are implicated in the pathogenesis of chronic non-communicable diseases (NCDs). Most studies over the past decades were designed to focus on the protective roles of soy in cardiovascular health, and had revealed that soy had favorable impacts on preventing the initiation and progression of cardiovascular disease (CVD) (6, 7). Our large prospective cohort studies have suggested that consumption of soy might reduce the risk of coronary heart disease (8). However, whether soy consumption is associated with risk of other major NCDs remains to be clarified.

Cancer and CVD potentially shared underlying biological mechanisms and common risk factors (9), indicating it seems plausible that soy intake may contribute to the decreased risk of cancer. Several prospective observational studies have been conducted to investigate soy intake in relation to risk of incident cancers, and yielded inconsistent results (10–12). Although some meta-analyses have reported inverse associations of soy with incidence of specific types of cancer, it is unclear whether the observed association still existed between soy and overall cancer (13–15). Furthermore, nutritional guidelines by the American Heart Association proposed that the cardiovascular protective effect offered by soy attributed to soy isoflavones and soy protein could be considered minimal (16). Little is known whether soy isoflavones or soy protein may impact the development of cancer. In addition, existing findings are largely lacking for the association of soy with prognosis of cancer.

Therefore, we conducted a meta-analysis of all available prospective cohort studies to assess the associations between intake of soy, soy isoflavones and soy protein and risk of cancer incidence as well as cancer mortality.

## Methods

The present study was conducted in accordance with the Meta-analysis of Observational Studies in Epidemiology (MOOSE) guidelines (17).

### Literature Search Strategy

The electronic databases PubMed, Embase, Web of Science, and Cochrane Library were utilized to systematically search up to 16 September 2021 for relevant prospective cohort studies examining the association between soy, soy isoflavones, soy protein and cancer incidence as well as cancer mortality. The following search items were included: (1) “soy” OR “soya” OR “bean” OR “soybean” OR “legume” OR “glycine max” OR “soy food” OR “tofu” OR “bean curd” OR “soymilk” OR “miso” OR “natto” OR “sufu” OR “tempeh” OR “genistein” OR “daidzein” OR “glycitein” OR “isoflavone” OR “phytoestrogen” OR “soy protein,” AND (2) “cancer” OR “neoplasm” OR “carcinoma” OR “tumor” OR “malignant,” AND (3) “morbidity” OR “incidence” OR “occurrence” OR “genesis” OR “occur” OR “mortality” OR “death” OR “fatal” OR “survival”. No restrictions were imposed with respect to publication language. Reference lists of retrieved articles and previous relevant reviews were scanned manually for pertinent studies. In addition, authors and experts of selected articles were contacted to identify any unpublished or ongoing studies that could fulfill inclusion criteria.

### Study Selection

Studies were considered to be eligible if they satisfied the following criteria: (1) studies with prospective cohort design were published as original articles; (2) the exposures of interest were dietary intake of soy, soy products (such as tofu, soymilk, bean sprouts, natto, miso and tempeh), soy isoflavones and soy protein; (3) the outcomes of interest were the incidence of cancer and cancer mortality; (4) relative risks (RRs) along with corresponding 95% confidence intervals (95% CIs) were reported or could be calculated with sufficient data. In case of multiple publications on the same population or subpopulation, the estimates from the most recent or most informative report were considered in the meta-analysis. Studies measuring concentrations of isoflavones or their metabolites in plasma or urine samples were excluded due to the difficulty in converting biological concentration into amount of soy isoflavones intake. Two investigators (YHF and ZFL) independently assessed full text of those selected publications to determine potentially relevant articles for inclusion; any discrepancies or uncertainties on eligibility were resolved by consulting a third reviewer (LM).

### Data Extraction and Quality Assessment

A standardized predesigned data collection form was applied to extract the following data from each included study: first author name, year of publication, geographical location, duration of follow-up, cohort name, sample size, mean age or age range at baseline, sex, dietary assessment method, categories of soy consumption, type of cancer, number of cases or deaths, method of endpoints identification, RRs (95% CI_S_) from the multivariable model, and potential covariates in the maximally adjusted model. Where studies reported RRs with different degrees of adjustment for other risk factors, the maximum adjusted estimate was prioritized. In addition, if an article included data from multiple cohorts, we considered the analysis for each cohort as an independent report and extracted data separately. Whenever necessary, the original study authors were contacted to obtain additional information that was not available in the online publications or Supplementary Materials.

Methodological quality of the included cohort study was assessed in accordance with the 9-star Newcastle-Ottawa scale (NOS), which focused on three domains: selection of participants (0–4 stars), comparability of cohorts (0–2 stars), and assessment of outcome (0–3 stars) (18). We assigned scores of 0–3, 4–6, and 7–9 for low, moderate, and high quality, respectively. Data extraction and quality assessment were conducted independently in a standardized manner by two researchers (YHF and JS), and the senior reviewer (LM) was involved to adjudicate any disagreements if necessary.

### Statistical Analysis

The pooled RRs were used as the common measures of association across studies. In order to take into account both within-study and between-study variability, random-effects models were applied to pool RRs and 95% CIs for the associations of soy, soy isoflavones and soy protein with cancer incidence and cancer mortality. For eligible studies that only reported stratified results by sex, cancer outcome or other variables, a within-study summary estimate was obtained using a fixed-effect model. Heterogeneity was quantified by Cochran's Q test (*P* < 0.10 for significance) and the I-square (*I*^2^) statistic with a value more than 50% signifying substantial heterogeneity (19). In further analysis, we explored the effect of individual soy foods in relation to cancer, including tofu, soy milk, miso and natto. Subgroup analysis and meta-regression were carried out by preset factors to explore the potential sources of heterogeneity, including gender (both, women or men), geographical location (the United States, Europe, or Asia), follow up periods (<10 y or ≥10 y), and adjustment for family history of cancer (yes or no). The potential dose-response associations were examined using the method proposed by Greenland and Longnecker, and only at least two studies with ≥3 exposures categories could be included (20). The mean or median soy, soy isoflavones and soy protein consumption per category was assigned to the corresponding RR estimate. For studies that continuous exposures were reported with a range, the midpoint of the upper and lower boundaries in each category was used to estimate median consumption. When the highest category was open-ended, the width of the adjacent interval was used to establish the highest cut-off value, and the midpoint of the lowest boundary and zero was defined as the amount of lowest quantile (21). For consistency, we converted different units (such as servings and times) to grams per day (g/d) according to standard conversions based on dietary guidelines and previous studies (22). Both non-linear and linear models were fitted and evaluated on the logarithm of the RR. Non-linear trends were examined using restricted cubic spline models with 3 knots at the 10, 50, and 90th percentiles, and the 2-stage generalized least-squares regression approach was used to estimate the linear dose-response slope (20). Sensitivity analysis by sequentially omitting individual study was performed to examine whether the pooled associations could be substantially affected by a single study, demonstrating the robustness of the findings. Publication bias was assessed by visual examination of funnel plots and statistically Egger's and Begg's Tests (23). The trim and fill method was applied to adjust for any observed publication bias via imputing additional studies. All statistical analyses were performed with Stata version 12.0 (Stata Corp, College Station, TX, USA). A two tailed *P* < 0.05 was deemed statistically significant, unless explicitly stated.

## Results

### Literature Search

**Figure 1:**
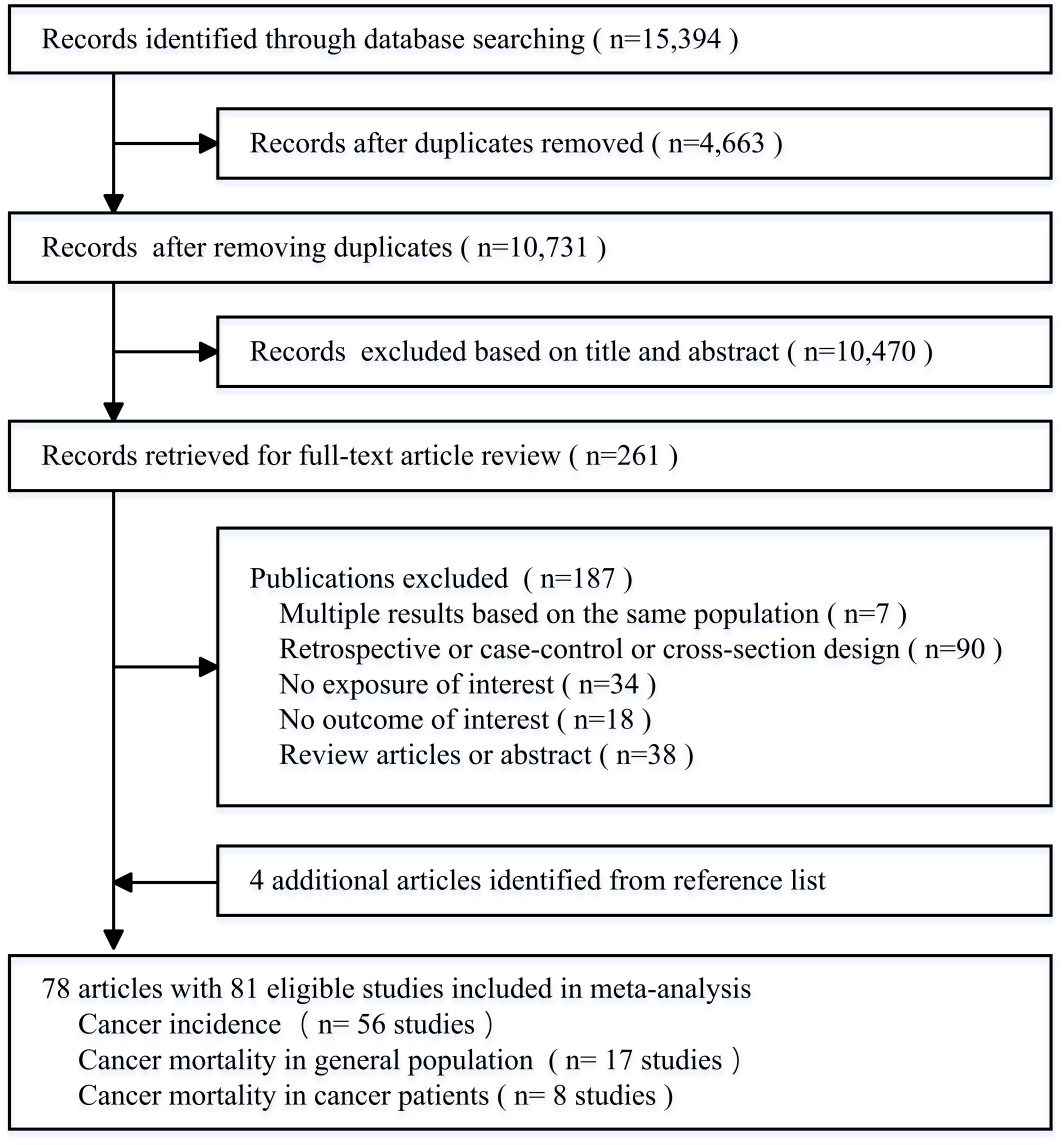
Flow diagram of study selection.

The search strategy yielded 10,731 records of potentially relevant articles from four databases after removing the duplicates, of which 261 full-text articles were retrieved for detailed evaluation after performing an initial screen of titles or abstracts. Hand searching of the bibliographic references of these articles identified 4 additional articles. Finally, 78 articles including 81 cohort studies were selected as appropriate for inclusion in the meta-analysis (Figure 1) (10–12, 15, 24–97).

### Study Characteristics

The main characteristics of the included studies are summarized in Supplementary Table 1. Among included studies, 51 studies were from Asia, 20 from the United States, and 10 studies were from Europe. The number of participants from each selected study varied from 1,210 to 477,312, for a total of about 4.15 million participants across studies. The study population in 32 studies comprised both men and women, 36 studies consisted of only women, and 13 studies involved men only. The follow-up duration ranged from 2 to 19.4 years. In most of the studies, information on dietary intake of soy, soy isoflavones and soy protein derived from food frequency questionnaire (FFQ). The endpoint of cancer was primarily confirmed by examination of medical records, cancer registries, or death certificates for included studies. The majority of studies reported risk estimates adjusted for age (*n* = 78), smoking (*n* = 58) and body mass index (*n* = 57). Many studies also adjusted for alcohol (*n* = 45), total energy intake (*n* = 43), physical activity (*n* = 36) and family history of cancer (*n* = 26). Results from the assessment of study quality showed that most studies (*n* = 74) were rated as high quality, and the others (*n* = 7) were deemed to be of medium quality (Supplementary Table 2).

### Intake of Soy, Soy Isoflavones and Soy Protein and Risk of Cancer Incidence

**Figure 2:**
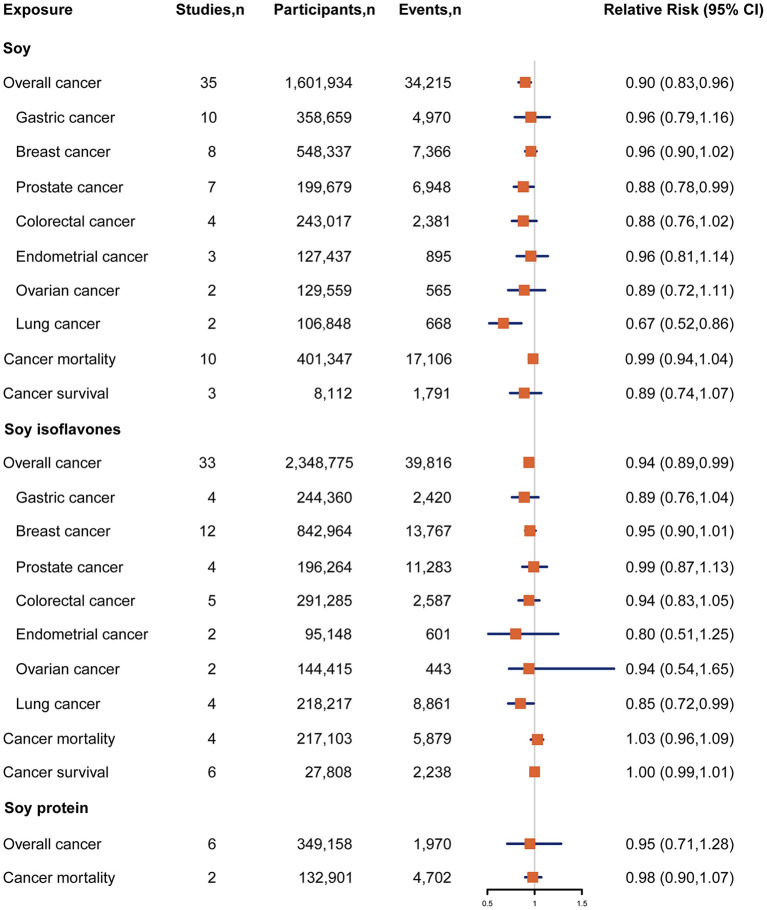
Forest plot for association between soy, soy isoflavones and soy protein intake and risk of cancer incidence and cancer mortality, expressed as comparison between highest and lowest categories of soy, soy isoflavones and soy protein intake. The size of the black squares reflects the relative statistical weight of study-specific estimate, horizontal lines indicate 95% CIs. The diamond indicates the pooled RR estimates with 95% CIs. CI, confidence interval; RR, relative risk.

**Figure 3:**
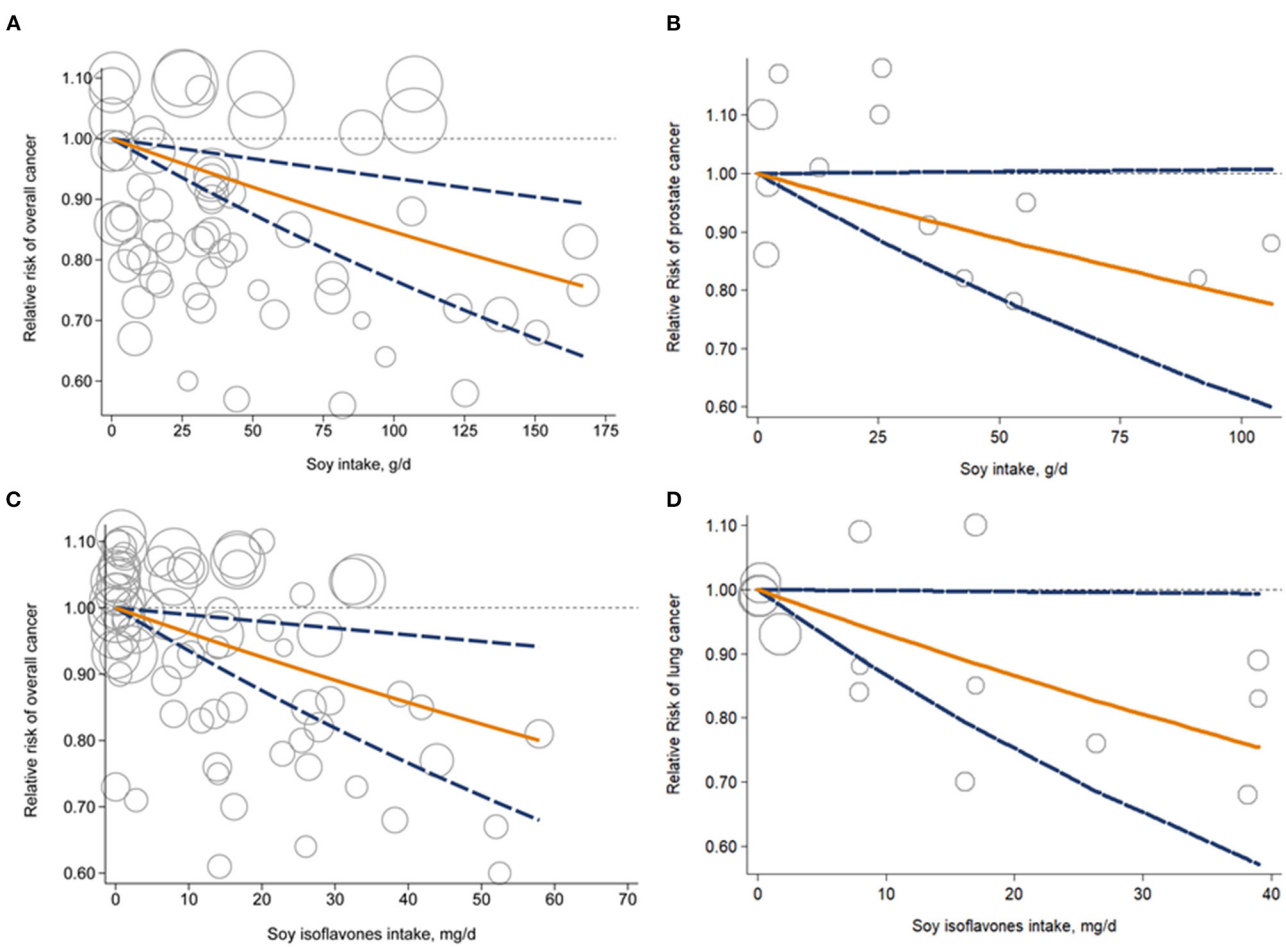
Dose-response analysis for liner association of soy intake with overall cancer risk **(A)** and prostate cancer risk **(B)**; soy isoflavones intake and overall cancer risk **(C)** and lung cancer risk **(D)**. Circles represent point estimates plotted over precision measures. Solid lines represent summary RRs; dashed lines are the corresponding 95%CIs. CI, confidence interval; RR, relative risk.

Forty seven studies examined the association between soy, soy isoflavones and soy protein intake and cancer risk (10–12, 15, 24–66). The pooled results showed that a higher intake of soy was significantly associated with a 10% reduced risk of overall cancer incidence when comparing extreme categories of soy intake (RR, 0.90; 95% CI, 0.83–0.96) (Figure 2). There was significant heterogeneity across the studies (*I*^2^ = 57.8%, *P*_heterogeneity_ <0.001). The dose-response analysis revealed that each increase of 25 g/d in soy intake significantly decreased the risk of overall cancer incidence by 4% (RR, 0.96; 95%CI, 0.94–0.98) (Figure 3A). In the stratified analyses across study and participant characteristics, inconsistencies in these variables did not significantly alter the shape of association between soy intake and risk of overall cancer incidence (Supplementary Table 3). In terms of soy isoflavones and soy protein, participants in the highest category of soy isoflavones intake had 6% (95% CI, 1–11%) lower risk of overall cancer incidence, compared with those in the lowest category (Figure 2). Evidence of substantial heterogeneity existed among studies (*I*^2^ = 52.6%, *P*_heterogeneity_ <0.001). No significant association between soy protein consumption and risk of overall cancer incidence was observed (RR, 0.95; 95% CI, 0.71–1.28). Each 10 mg/d increment of soy isoflavones intake was significantly associated with a 4% lower risk of overall cancer incidence (RR, 0.96; 95% CI, 0.94–0.99) in the dose-response analysis (Figure 3C). The association between soy isoflavones intake and risk of overall cancer incidence did not differ substantially by characteristics of study and participant examined (Supplementary Table 4).

### Intake of Soy, Soy Isoflavones and Soy Protein and Risk of Site-Specific Cancers

Associations between intake of soy, soy isoflavones and soy protein and risk of specific cancer sites were investigated in 54 studies {breast cancer (*n* = 15) (24–26, 29, 33, 38, 41, 46, 48, 51, 53, 56, 59, 61, 67), gastric cancer (*n* = 10) (31, 32, 36, 37, 54, 62, 64, 65, 68), prostate cancer (*n* = 9) (28, 34, 47, 50, 55, 57, 63, 66, 69), colorectal cancer (*n* = 5) (30, 43, 44, 52, 70), bladder cancer (*n* = 4) (11, 35, 58, 60), lung cancer (*n* = 4) (15, 42, 45, 71), endometrial cancer (*n* = 3) (26, 39, 72), ovarian cancer (*n* = 3) (26, 40, 49), cervical cancer (*n* = 1) (27), pancreatic cancer (*n* = 1) (73), liver cancer (*n* = 1) (74)}. Participants in the highest category of soy intake had a 33% lower risk of lung cancer compared with those in the lowest category (RR, 0.67; 95% CI, 0.52–0.86) (Figure 2). A similar inverse association was also observed between soy intake and risk of prostate cancer (RR, 0.88; 95% CI, 0.78–0.99), with low heterogeneity detected among studies (*I*^2^ = 12.3%; *P*_heterogeneity_ = 0.34) (Figure 2). The dose-response analysis showed that each 25 g/d increment of soy intake was marginally associated with 6% (RR, 0.94; 95% CI, 0.89–1.00) lower risk of prostate cancer (Figure 3B). Regarding the association of soy isoflavones with lung cancer, the pooled RR of lung cancer was 0.85 (95% CI, 0.72–0.99) when comparing the extreme categories of soy isoflavones intake. Association of soy protein intake and risk of most individual cancers could not be evaluated due to the limited number of studies. The dose-response analysis revealed a significant 6% decrease in risk of lung cancer for a 10 mg/d increase in soy isoflavones intake (RR, 0.94; 95% CI, 0.89–0.98) (Figure 3D).

### Intake of Soy, Soy Isoflavones and Soy Protein and Risk of Cancer Mortality in General Population

The associations between soy, soy isoflavones and soy protein intake and risk of cancer mortality in general population were reported in 17 studies (75–90). Among these studies, most did not report a significant inverse association except one study (88). The pooled RR for cancer mortality comparing the extreme categories of soy intake was 0.99 (95% CI, 0.94–1.04) (Figure 2). No heterogeneity was found between these studies (*I*^2^ = 0.0%, *P*_heterogeneity_ = 0.64). Both soy isoflavones and soy protein intake were not associated with risk of cancer mortality, with a pooled RR of 1.03 (95% CI, 0.96–1.09; *I*^2^ = 0.0, *P*_heterogeneity_ = 0.46) and 0.98 (95% CI, 0.90–1.07; *I*^2^ = 0.0%, *P*_heterogeneity_ =0.81), respectively (Figure 2).

### Intake of Soy, Soy Isoflavones and Risk of Cancer Mortality in Cancer Patients

Eight studies were included in the analysis of soy and soy isoflavones intake with risk of cancer mortality in cancer patients (i.e., cancer survival) (91–97). All these studies reported non-significant associations. The pooled results showed that a higher intake of soy was not significantly associated with a lower risk of cancer mortality in cancer patients when comparing extreme categories of soy intake (RR, 0.89; 95% CI, 0.74–1.07) (Figure 2). Little evidence of heterogeneity was found (*I*^2^ = 0.0%, *P*_heterogeneity_ = 0.39). No statistically significant association was also found between soy isoflavones intake and risk of cancer mortality among cancer patients (RR, 1.00; 95% CI, 0.99–1.01) (Figure 2).

### Sensitivity Analysis and Publication Bias

Sensitivity analyses showed that no individual study excluded changed the combined RR substantially between soy, soy isoflavones and soy protein and any of the outcomes. The Egger's (*P* > 0.05) or Begg's (*P* > 0.05) did not indicate the presence of publication bias for most associations examined in the current meta-analysis, except for the associations between soy protein intake and risk of overall cancer incidence (*P* = 0.03 for Egger's test and *P* = 0.06 for Begg's test); soy intake and risk of prostate cancer (*P* = 0.03 for Egger's test and *P* = 0.07 for Begg's test). Furthermore, the trim and fill correction procedures indicated that the pooled RRs were not affected by publication bias (data not shown).

## Discussion

The present meta-analysis suggested that increasing intake of soy was significantly associated with a lower risk of overall cancer incidence as well as site-specific cancers, particularly lung and prostate cancer. Similar risk reduction was also observed for intake of soy isoflavones, rather than soy protein. In contrast, no significant association was detected for dietary intake of soy, soy isoflavones and soy protein in relation to cancer mortality for both general population and cancer patients.

Numerous prospective observational studies had been conducted to examine the association of soy intake with cancer risk. In a cohort of 30,817 Japanese men and women, a significant decreased risk of bladder cancer was seen among men who had highest category of soy intake compared with those in the lowest category (11). Among 68,412 women free of cancer from Shanghai Women's Health Study, each 5 g/d increment in soy intake was associated with an 8% reduction in colorectal cancer risk for postmenopausal women (15). However, in the Women's Health Study consisting of 38,408 US women, higher intake of tofu was not significantly associated with total cancer as well as site-specific cancer risk during over 10-year follow up period (10). Although previous meta-analyses reported that soy consumption was inversely associated with 15–37% lower risks of cancer in different sites, such as stomach, prostate and lung cancer (13–15), no meta-analysis has summarized the association for soy intake in relation to overall cancer risk. Taking cancer together is a matter of vital importance for a more comprehensive way to assess the prevention of cancer morbidity and mortality for both general population and cancer patients. Our results showed that a higher intake of soy was associated with a decreased risk of overall cancer incidence. Furthermore, this result was also supported by our dose-response finding in which the risk of overall cancer decreased with increment in soy intake. Of note, the direction of the inverse association between soy intake and risk of cancer was generally similar to those in results of soy isoflavones, suggesting that the protective effects of soy on cancer may be primarily attributed to soy isoflavones.

Several biological mechanisms have been proposed for the potential health benefits of soy, especially isoflavones on preventing cancer, involving an increased antioxidant effect and reduced inflammation. It has been well-established that elevated levels of reactive oxygen species could damage DNA, drive gene mutations, and contribute to an increase in cell proliferation or a decrease in cell apoptosis, subsequently leading to mutagenesis and carcinogenesis. Soy and soy isoflavones have been suggested to counteract oxidative stress by activation of nuclear factor erythroid 2-related factor 2 (Nrf2) (4, 98) and modulation of genes expression involved in cell proliferation and cell apoptosis (99). In a 1,2-dimethyl hydrazine-induced colon cancer model in Wistar rats, genistein treatment for 6 weeks increased Nrf2 and hemoxygenase-1 protein expression and maintained glutathione levels (100). *In vivo*, isoflavones could enhance glutathione S-transferase and quinone reductase activity in SD rats fed soy diet for 2 weeks (101). Using human MCF-7 breast cancer cell line, Jin et al. revealed that treatment with daidzein for 24 h could induce cell apoptosis via down-regulation of B cell leukemia/lymphoma2 (BCL2) expression and up-regulation of BCL2-associated X protein expression in the mitochondrial caspase-dependent cell death pathway (102). Takahashi et al. also reported isoflavones exhibited an inhibitory effect on mitogen-activated protein kinase phosphatase 2 in human prostate cancer cell LNCaP, which blocked mitogenic signal transduction (103). Moreover, soy isoflavones were thought to suppress the expression of inflammatory mediators and block sustained damage-induced cellular proliferation through activation of nuclear factor kappa-B (NF-κB) (104, 105). In activated macrophage-Like RAW 264.7 cells, 8-Hydroxydaidzein treatment diminished expression of nitric oxide synthase, cyclooxygenase-2 and tumor necrosis factor-&alpha by inactivating the transcriptional activities of NF-κB and activator protein 1 (106). Also, a 5-month diet with genistein could downregulate pro-inflammatory responses and ameliorate liver damage by phosphorylation of adenosine monophosphate-activated protein kinase for C57BL/6 mices injected with diethylnitrosamine (107). As a critical inhibitor of protein-tyrosine kinases (PTK), isoflavones may also exert anti-mitotic and anti-angiogenic effects by inhibiting endothelial cell proliferation, migration, and capillary structure formation in response to vascular endothelial growth factor (VEGF) (108). Yu et al. found that the genistein could interrupt VEGF-stimulated human umbilical vein endothelial cells activation by inhibition of PTK activity and decreasing matrix metalloproteinases production that promoted angiogenesis (109).

In contrast with the favorable impact of soy on cancer incidence, our findings suggested that soy intake was not associated with the risk of cancer mortality for both general population and cancer patients. The discrepancies appear to be explained by the fact that cancer incidence and cancer mortality are two very distinct outcomes, with cancer mortality being greatly determined by the treatment approaches individual receives (110). It is well-known that cancer treatments induce many adverse symptoms including nausea/vomiting, impairment of taste and smell and bowel changes, which could interfere with the ability to eat, digest, or absorb soy (111). In a comparison of blood daidzein levels between prostate cancer patients and controls, the proportion of equol producers was significantly lower among prostate cancer patients, indicating that conversion of daidzein into equol is regulated by isoflavones ingestion and intestinal metabolism (112). Alternatively, approximately three out of four cancer patients suffer from at least one coexisting chronic disease, sharing biological mechanisms and common risk factors with cancer. Most comorbidities may alter the risk of cancer mortality because multiple pathological processes combined occur simultaneously (113). Lane et al. found that 29% of 537 renal tumor patients who received active treatment, died mostly of cardiovascular causes compared to 4% of those who died of cancer (114). Consistently, in a recent meta-analysis of 28 prospective and retrospective cohort studies on breast, lung, prostate, and colorectal cancer, and glioma, increased dietary intake of isoflavone was only significantly associated with risk of total mortality, rather than cancer-specific mortality in patients with breast cancer, which further reflected the potential prevalence of coexisting chronic diseases in cancer patients (115). Furthermore, it should be noted that an increased prevalence of competing risk factors, for example the adverse effects of obesity, physical inactivity, smoking, and high alcohol consumption, may result in the reduction of the proportional effect of dietary soy intake.

The potential limitations of the present meta-analysis are worth discussion in interpreting our findings. First, although most studies controlled for a wide range of potential socio-demographic, lifestyle, and dietary factors, the possibility of residual confounding by unmeasured or imprecisely measured factors cannot be completely ruled out, which is inherent to observational studies. Second, because self-reported dietary intake through FFQ is subject to bias, some measurement errors or misclassifications in soy intake assessment are inevitable. By prospectively collecting diet data before cancer occurrence or cancer death in most studies, any misclassification of soy intake would be non-differential and thus tend to bias results toward the null, resulting in the underestimation of true associations. Third, the observed relationship may be influenced by synergistic or additive effects of soy isoflavones and soy protein with other nutrients and non-nutrient constituents from soy. More future studies with larger sample sizes are warranted to examine whether these interactions may modify the associations between soy, soy isoflavones and soy protein intake and cancer risk. Fourth, different methods used in the cooking and processing of soy might influence the nutraceutical values of soy and bioavailability of isoflavones and soy protein. In our study, whether the individual soy foods were non-fermented or fermented did not exert significant changes in risk of cancer. Likewise, an intervention trial conducted among free-living individuals showed that consumption of different types of soy products did not alter urinary isoflavone excretion, indicating that the cooking or preparation methods might not appreciably affect the associations observed (116). Fifth, limited data was available for the association between soy protein and cancer despite a comprehensive literature research and inclusion of a considerable number of studies, more future large-scale prospective studies, particularly evaluating soy protein, are warranted to clarify the effects of soy protein on cancer incidence and cancer mortality. Finally, publication bias could be of concern in all meta-analyses. Although there was no evidence of significant publication bias for the outcomes in our study, the possibility of the undetected selection bias introduced because of exclusion of gray literature and unpublished data cannot be fully eliminated.

## Conclusions

Our findings of the current meta-analysis suggested that higher intake of soy and soy isoflavones were inversely associated with risk of cancer in a dose-response manner, indicating that the beneficial role of soy against cancer may be primarily attributed to soy isoflavones. These findings support recommendations to include soy as part of a healthy dietary pattern for the prevention of cancer.

## Data Availability Statement

The original contributions presented in the study are included in the article/Supplementary Material, further inquiries can be directed to the corresponding author/s.

## Author Contributions

LM, WZ, and LK conceived the study and formulated an analytical plan and supervised the study. YF and ZL designed the search strategy, performed the literature search, and screened studies for eligibility. YF, HJ, and XS extracted the data. YF and JS contributed to the study quality assessment. YF, HJ, SL, and JZ performed the statistical analysis. YF, MW, LM, WZ, and LK drafted the original manuscript and all other authors reviewed and revised the draft of the manuscript. All authors approved the final manuscript for submission.

## Funding

This study was partially supported by grants from the National Natural Science Foundation of China (NSFC-82022062; NSFC-81973025; NSFC-81473059); Nutrition Science Research Foundation of BY-HEALTH (TY0181101); the Natural Science Foundation of Shaanxi Province of China (2017JM8041); New-star Plan of Science and Technology of Shaanxi Province (2015LJXX-07); the Nutrition Research Foundation Fund of the Chinese Nutrition Society-DSM Special Research Foundation (CNSDSM2016-041); the Fundamental Research Funds for the Central Universities (qngz2016004; xzy032019008). The funders had no role in the study design, implementation, analysis, decision to publish, or reparation of the manuscript.

## Conflict of Interest

The authors declare that the research was conducted in the absence of any commercial or financial relationships that could be construed as a potential conflict of interest.

## Publisher's Note

All claims expressed in this article are solely those of the authors and do not necessarily represent those of their affiliated organizations, or those of the publisher, the editors and the reviewers. Any product that may be evaluated in this article, or claim that may be made by its manufacturer, is not guaranteed or endorsed by the publisher.
