# Supplementary material for: Intake of Soy, Soy Isoflavones and Soy Protein and Risk of Cancer Incidence and Mortality
Source: Front Nutr. 2022 Mar 4;9:847421. doi: 10.3389/fnut.2022.847421 (PMC8931954; doi:10.3389/fnut.2022.847421)
Supplement: Supplementary file 1 [file Data_Sheet_1.docx]

Supplementary Material

**Table S1 ǀ** General characteristics of included cohort studies (n=81)

| **author,**  **publication year,**  **country ^(Ref)^** | **Cohort name** | **Follow-**  **up period**  **(year)** | **Participants** | **Health status** | **Men (%)** | **Exposure assessment** | **Exposure** | **Outcome type**  **(cases)** | **Ascertainment**  **method** | **Adjustment for confounding factors** | **Study quality*** |
| --- | --- | --- | --- | --- | --- | --- | --- | --- | --- | --- | --- |
| Abe et al, 2021, Japan^73^ | Japan Public Health Center Study | 1995-2013  (15.3) | 75,089 men and women aged  40-69y | General population | 46.2 | 138-item validated FFQ | Total soy foods,  non-fermented, fermented, tofu, soymilk, fried tofu, dried tofu  miso, natto | Liver cancer incidence  (534) | Medical records,  cancer registries,  death certificates | Age, area, smoking, alcohol, BMI, history of type 2 diabetes, hepatitis C virus, HBsAg, menopausal status, coffee intake | 9 |
| Ho et al, 2021, China^90^ | Hong Kong Breast Cancer Survival  Study | 2011-2018  (6.0) | 1,460 women  aged  25-79y | Breast cancer  patients | 0.0 | 107-item validated FFQ | Soy isoflavone | Breast cancer  mortality  (115) | Medical records, computerized clinical management system,  death registries | Age, education, menopausal status, cancer stage, comorbidity, ER status, PR status, HER2 status, hormonal therapy, radio therapy | 8 |
| Nozue et al, 2021, Japan^12^ | Japan Public Health Center Study | 1995-2012  (13.1) | 79,648 men and women aged  45-74y | General population | 46.3 | 138-item validated FFQ | Total soy products, non-fermented, fermented, miso, natto,  soy isoflavone | Total cancer incidence (9,972) | Patient notification,  cancer registries | Age, area, smoking, alcohol, BMI, total energy intake,  metabolic equivalents hours, menopausal status, coffee intake, intake of vegetable, fruit, fish, green tea | 9 |
| Shirabe et al, 2021, Japan^66^ | Japan Public Health Center Study | 1990-2013  (15.5) | 47,614 women aged  45-74y | General population | 0.0 | 138-item validated FFQ | Soy foods, non-fermented,  fermented, tofu, soymilk, fried tofu, dried tofu, miso, natto, soy isoflavone | Breast cancer incidence (825) | Patient notification,  cancer registries | Age, area, smoking, number of cigarettes smoked per day, alcohol, BMI, height, total energy intake, physical activity, past history of diabetes, family history, received mammography, age at menarche, age at first birth, number of deliveries, menopausal age, hormone use, breast feeding | 9 |
| Fraser et al, 2020, United  States^23^ | Adventist Health Study-2 | 2002-2012  (7.9) | 52,795 women  aged  57.1y  (mean) | General population | 0.0 | 68-item validated FFQ | Soy beans and tofu, soy milk,  soy isoﬂavone | Breast cancer  incidence  (1,057) | Cancer registries | Race, smoking, alcohol, BMI, physical activity, family history of breast cancer, time since mammography, menopausal status, oral contraceptive use, nulliparous, BMI*menopausal status, age at menarche, age at menopause, menopause, estrogen replacement therapy and progestogen therapy among postmenopausal women, duration of breastfeeding, number of children, age at first childbirth among parous women, dairy energy, intake of calcium, unprocessed and processed red meats, poultry, fish, seeds and nuts; supplement isoflavone | 7 |
| Katagiri et al, 2020, Japan^74^ | Japan Public Health Center Study | 1995-2012  (14.8) | 92,915 men and women  aged  45-74y | General population | 46.0 | 138-item validated FFQ | Total Soy products,  non-fermented, fermented, tofu miso, natto | Total cancer mortality  (5,137) | Residential registry,  death certificates | Age, area, smoking, alcohol, BMI, total energy intake, physical activity, history of diabetes, taking drugs for diabetes, taking antihypertensives, health check-up, coffee intake, intake of vegetable, fruit, green tea, fish, meat | 9 |
| Minami et al, 2020, Japan^91^ | NA | 1997-2016  (6.5) | 1,931  men and women  aged  ≥30y | Gastric cancer  patients | 69.9 | 40-item validated FFQ | Soy foods, miso soup | Gastric cancer mortality  (512) | Cancer registries | Age, sex, occupation, smoking, alcohol, BMI, total energy intake, year of diagnosis, stage, histological type, referral status, family history of gastric cancer, comorbidity, curative resection, chemotherapy | 8 |
| Minami et al, 2020, Japan^91^ | NA | 1997-2016  (7.1) | 793  men and women  aged  ≥30y | Colon cancer  patients | 54.0 | 40-item  validated FFQ | Soy foods, miso soup | Colon cancer  mortality  (187) | Cancer registries | Age, sex, occupation, smoking, alcohol, BMI, total energy intake, year of diagnosis, stage, histological type, referral status, family history of gastric cancer, comorbidity, curative resection, chemotherapy | 8 |
| Minami et al, 2020, Japan^91^ | NA | 1997-2016  (6.2) | 510  men and women  aged  ≥30y | Rectum cancer  patients | 51.4 | 40-item validated FFQ | Soy foods, miso soup | Rectum cancer mortality  (135) | Cancer registries | Age, sex, occupation, smoking, alcohol, BMI, total energy intake, year of diagnosis, stage, histological type, referral status, family history of gastric cancer, comorbidity, curative resection, chemotherapy | 8 |
| Sawada et al,  2020, Japan^75^ | Japan Public Health Center Study | 1995-2016  (16.9) | 43,580 men  aged  45-74y | General population | 100.0 | 138-item validated FFQ | Soy foods, tofu, miso, natto,  soy isoflavone | Prostate cancer  mortality  (221) | National Vital Statistics | Age, area, smoking, alcohol, BMI, physical activity, history of diabetes mellitus, screening, coffee intake, intake of vegetable, fruit, green tea | 9 |
| Wei et al, 2020, China^24^ | China Kadoorie Biobank Study | 2004-2016  (10.0) | 300,852  women  aged  30-79y | General population | 0.0 | validated FFQ | Soy,  soy isoflavone | Breast cancer incidence  (2,289) | Disease and death registries,  national health insurance system | Age, area, education, family income, smoking, alcohol, BMI, height, total energy intake, physical activity, family history of cancer, age at menarche, parity, duration of breastfeeding, menopausal status, age at menopause, use of oral contraceptives, intake of fresh vegetable, preserved vegetable, fresh fruit, fish, red meat, poultry, dairy products | 9 |
| Yamagiwa et al, 2020, Japan^72^ | Japan Public Health Center Study | 1995-2013  (16.9) | 90,185 men and women aged  40-69y | General population | 46.5 | 138-item validated FFQ | Total soy foods, non-fermented, fermented, tofu, miso, natto, genistein | Pancreatic cancer incidence  (577) | Medical records,  cancer registries,  death certificates | Age, sex, smoking, alcohol, BMI, total energy intake, physical activity, history of diabetes mellitus, family history of pancreatic cancer, coffee intake, intake of vegetable, fruit, fish, meat | 9 |
| Dunneram et al, 2019, United Kingdom^25^ | UK Women’s Cohort Study | 1995-2016  (18.0) | 32,228  women aged  35-69y | General population | 0.0 | 217-item validated FFQ | Soybean products | Breast cancer incidence  (1,822) | National Health Service Central Register | Age, social class, smoking, alcohol, physical activity, duration of breastfeeding, menopausal status | 8 |
| Dunneram et al, 2019, United Kingdom^25^ | UK Women’s Cohort Study | 1995-2016  (18.0) | 32,289 women aged  35-69y | General population | 0.0 | 217-item validated FFQ | Soybean products | Endometrial cancer incidence  (294) | National Health Service Central Register | Age, social class, smoking, alcohol, physical activity, history of diabetes, history of hypertension, duration of breastfeeding, menopausal status | 8 |
| Dunneram et al, 2019, United Kingdom^25^ | UK Women’s Cohort Study | 1995-2016  (18.0) | 32,284 women aged  35-69y | General population | 0.0 | 217-item validated FFQ | Soybean products | Ovarian cancer  incidence  (285) | National Health Service Central Register | Age, social class, smoking, alcohol, physical activity, duration of breastfeeding, menopausal status | 8 |
| Paul et al, 2019, Singapore^26^ | Singapore Chinese  Health Study | 1993-2013  (16.7) | 30,744  women aged  45-74y | General population | 0.0 | 165-item validated FFQ | Soy foods,  soy isoflavone | Cervical cancer incidence  (312) | Cancer registries,  Singapore registry of births and deaths | Age, dialect group, year of interview, education, smoking, total energy intake, duration of oral contraceptive use, history of pap-based test, parity, menopausal status | 8 |
| Reger et al, 2018, United States^27^ | Prostate, Lung, Colorectal and Ovarian Cancer  Screening Trial | 1993-2001  (11.5) | 27,004 men aged  55-74y | General population | 100.0 | 137-item FFQ | Total soy isoflavones,  genistein, daidzein,  glycitein | Prostate cancer incidence  (2,598) | Prostate biopsy | Age, race, smoking, alcohol, BMI, family history of prostate cancer | 9 |
| Wada et al, 2018, Japan^11^ | Takayama Study | 1992-2008  (13.6) | 30,817 men and women aged  ≥35y | General population | 46.2 | 169-item validated FFQ | Soy foods,  soy isoflavone | Bladder cancer  incidence  (161) | Cancer registries,  death certificates | Age, education, smoking, alcohol, BMI, physical activity,  menopausal status | 9 |
| Tang et al, 2017, China^76^ | Shanghai Women’s Health Study | 1997-2013  (14.7) | 73,044 women  aged  40-70y | General population | 0.0 | 77-item validated FFQ | Soy foods,  non-fermented, soy isoflavone  soy protein | Total cancer mortality  (2,580) | Cancer and vital statistics registry | Age, income, education, occupation, smoking, alcohol, BMI, total energy intake, physical activity, history of diabetes, family history of cancer, menopausal status, intake of vegetable, fruit and red meat | 9 |
| Tang et al, 2017, China^76^ | Shanghai Men’s Health Study | 2002-2013  (9.2) | 59,857  men aged  40-74y | General population | 100.0 | 81-item validated FFQ | Soy foods,  non-fermented, soy isoflavone  soy protein | Total cancer mortality  (2,122) | Cancer and vital statistics registry | Age, income, education, occupation, smoking, alcohol, BMI, total energy intake, physical activity, history of diabetes, family history of cancer, intake of vegetable, fruit and red meat | 9 |
| Baglia et al, 2016, China^28^ | Shanghai Women’s Health Study | 1996-2015  (13.2) | 70,578 women aged  40-70y | General population | 0.0 | 77-item validated FFQ | Soy isoflavone,  soy protein | Breast cancer incidence (1,034) | Cancer registries | Age, education, season of recruitment, BMI, total energy intake, physical activity, family history of breast cancer, age at ﬁrst live birth, menopause | 9 |
| Hedelin et al, 2016, Sweden^29^ | Women’s Lifestyle and Health  Study | 1991-2010  (19.0) | 48,268 women aged  30-49y | General population | 0.0 | 80-item validated FFQ | Soy isoflavone | Colorectal cancer incidence  (206) | Cancer registries, population register | Age, education, smoking, alcohol, BMI, total energy intake, physical activity, intake of vegetable, fruit, ﬁsh, processed meat, saturated fat, vitamin D, ﬁber and individual phytoestrogen | 8 |
| Leo et al, 2016, United  States^92^ | Multiethnic Cohort Study | 1993-2010  (4.5) | 2,339  men and women  aged  45-75y | NHL patients | 53.0 | Over-180 item  validated  FFQ | Soy foods | Non-Hodgkin lymphoma mortality  (903) | California  and Hawaii vital record, national death | Age, sex, education, smoking, alcohol, BMI, age at diagnosis, 5-year survival, comorbidity, NHL type, SEER stage chemotherapy, radiotherapy, surgery, immunotherapy, steroid treatment | 7 |
| Umesawa et al, 2016, Japan^30^ | Japan Collaborative Cohort Study | 1988-2009  (14.3) | 40,729 men and women aged  40-79y | General population | 38.6 | 33-item validated FFQ | Miso soup | Gastric cancer incidence (787) | Medical record,  cancer registries,  death certificates | Age, sex, education, smoking, alcohol, BMI, physical activity, family history of gastric cancer, perceived mental stress, intake of vegetable, fruit | 9 |
| Budhathoki et al, 2015, Japan^71^ | Japan Public Health Center Study | 1995-2009 (12.1) | 49,121 women aged  45-74y | General population | 0.0 | 138-item validated FFQ | Soy foods, tofu, miso soup,  soy isoflavone | Endometrial cancer  incidence  (112) | Cancer registries,  death certificates | Age, area, smoking, alcohol,  BMI, physical activity, past history of diabetes mellitus and cancer, age at menarche, exogenous hormone use, number of deliveries, menopausal status, age at menopause for postmenopausal women, coffee intake | 9 |
| Kyrø et al, 2015, Europe^93^ | European Prospective Investigation into Cancer and Nutrition | 2004-2010  (6.3) | 11,782 women  aged  46-73y | Breast cancer  patients | 0.0 | 178-item  validated FFQ | Soy isoflavone | Breast cancer  mortality (753) | Mortality registry, health insurance record,  cancer and pathology registry | 5-year age group, country, smoking, alcohol, BMI, physical activity, HRT use, schooling, ER status, cancer stage, grading of tumor | 7 |
| Wada et al, 2015, Japan^31^ | Takayama Study | 1992-2008  (13.6) | 30,792 men and women  aged  ≥35y | General population | 46.2 | 169-item validated FFQ | Soy foods,  non-fermented,  fermented,  soy isoflavone | Gastric cancer incidence (678) | Cancer registries,  death certificates | Age, education, smoking, alcohol, BMI, physical activity, menopausal status, salt intake | 9 |
| Yamasaki et al,2015, Japan^77^ | Jichi Medical School Cohort Study | 1992-2005  (11.8) | 11,066 men and women  aged  19-93y | General population | 38.9 | 30-item validated FFQ | Soy,  soy products | Total cancer mortality  (346) | Death certificates | Age, education, smoking, alcohol, BMI, hypertension, diabetes, high-density lipoprotein cholesterol, menopause status | 8 |
| Morimoto et al, 2014,  United  States^22^ | Multiethnic Cohort Study | 1993-2007  (13.0) | 84,450 women  aged  45-75y | General population | 0.0 | Over-180 item  validated  FFQ | Soy isoflavone | Breast cancer incidence  (4,769) | Cancer registries | Age, education, race, smoking, alcohol, BMI, total energy  intake, family history of breast cancer diabetes, hypertension, age at menarche, age at ﬁrst live birth, parity, menopausal status, oral contraceptive use, menopausal hormone use | 8 |
| Wang et al, 2014, United  States^33^ | CPS II  Nutrition Cohort  Study | 1999-2009  (7.8) | 43,268 men aged 50-74y | General population | 100.0 | 152-item validated FFQ | Soy isoflavone | Prostate cancer  incidence  (3,974) | Medical record,  cancer registries,  National  Death Index | Age, race, smoking, BMI, total energy intake, family history of prostate cancer, history of diabetes, aspirin use, history of prostate-specific antigen screening | 9 |
| Zamora-Ros et al, 2014, Europe^34^ | European Prospective Investigation into Cancer and Nutrition | 1991-2010  (11.0) | 477,312 men and women  aged  35-70y | General population | 29.8 | 178-item  validated FFQ | Soy isoflavone | Bladder cancer  incidence  (1,575) | Cancer registries | Age, sex, education, center, smoking, alcohol, BMI, total energy intake, physical activity | 9 |
| Conroy et al,  2013, United  States^94^ | Multiethnic Cohort Study | 1993-2007  (6.2) | 3,842 women  aged  45-75y | Breast cancer  patients | 0.0 | Over-180 item  validated  FFQ | Soy products,  soy isoflavone | Breast cancer  mortality  (376) | Death certificates, National Death Index | Age at diagnosis, race, smoking, BMI, total energy intake, stage, hormone receptor status, treatment, cardiovascular comorbidity, history of diabetes, years between cohort entry and diagnosis | 8 |
| Kweon et al, 2013, China^35^ | Shanghai Women’s Health Study | 1996-2010  (14.0) | 70,446 women  aged  40-70y | General population | 0.0 | 77-item validated FFQ | Non-fermented soy foods, tofu, soy milk, dry bean, fresh bean, bean sprout, other soy foods, soy isoflavone, soy protein | Gastric cancer incidence  (282) | Cancer registries,  death certiﬁcates | Age, family income, smoking, ever smoke, alcohol, BMI, total energy intake, metabolic equivalents hours, chronic gastritis history, family gastric cancer history, born in urban Shanghai, intake of red meat, vegetable, sodium, fruit (excluding watermelon) | 9 |
| Kweon et al, 2013, China^35^ | Shanghai Men’s Health Study | 2002-2010  (8.0) | 58,241 men aged  40-74y | General population | 100.0 | 81-item  validated FFQ | Non-fermented soy foods, tofu, soy milk, dry bean, fresh bean, bean sprout, other soy foods, soy isoflavone, soy protein | Gastric cancer incidence  (211) | Cancer registries,  death certiﬁcates | Age, smoking, alcohol, ever smoke, family income, BMI, total energy intake, metabolic equivalents hours, chronic gastritis history, family gastric cancer history, born in urban Shanghai, intake of vegetable, fruit (excluding watermelon), red meat, sodium | 9 |
| Ko et al, 2013, Korea^36^ | Korean Multi-Center Cancer Cohort Study | 1993-2008  (8.5) | 9,724  men and women  aged  30-90y | General population | 38.2 | FFQ | Soybean/tofu,  soybean paste | Gastric cancer incidence  (166) | Cancer registries,  death certiﬁcates | Age, sex, area, smoking, alcohol, BMI | 8 |
| Zamora-Ros et al, 2013, Spain^78^ | European Prospective Investigation into Cancer and Nutrition -Spain Study | 1992-2009  (13.6) | 40,622 men and women  aged  20-69y | General population | 38.0 | 178-item  validated FFQ | Soy isoflavone | Total cancer mortality  (956) | Regional mortality registry, National Death Index | Age, sex, education, center, smoking, alcohol, BMI, total energy intake, physical activity, intake of vitamin C and fiber | 8 |
| Wada et al, 2013, Japan^37^ | Takayama Study | 1992-2008  (14.0) | 15,607  women aged  ≥35y | General population | 0.0 | 169-item validated FFQ | Soy,  soy isoflavone | Breast cancer incidence (172) | Cancer registries,  death certificates | Age, education, smoking, alcohol, BMI, physical activity, age at menarche, age at first delivery, menopausal status, parity number, HRT use | 9 |
| Hara et al, 2012, Japan^67^ | Japan Public Health Center Study | 1995-2006  (9.5) | 84,881 men and women  aged  45-74y | General population | 46.6 | 138-item validated FFQ | Soy foods, miso soup,  soy isoflavone | Gastric cancer incidence  (1,249) | Cancer registries | Age, area, smoking, alcohol, BMI, total energy intake, family history of gastric cancer, intake of vegetable, fruit, ﬁsh, salt | 9 |
| Nechuta et al, 2012, United  States^95^ | Life After  Cancer Epidemiology Study, Women’s  Healthy Eating & Living Study | 1991-2006  (7.4) | 4,658 women  aged  ＞18y | Breast cancer  patients | 0.0 | Validated FFQ | Soy isoflavone | Breast cancer  mortality  (476) | Medical records,  death index | Age at diagnosis, education, race-ethnicity, smoking, BMI, physical activity, ER/PR status, TNM stage, chemotherapy, radiotherapy, hormonal therapy, parity, menopausal status, intake of cruciferous vegetable, study | 8 |
| Nechuta et al, 2012, China^95^ | Shanghai  Breast Cancer Survival Study | 1991-2006  (7.4) | 4,856  women  aged  25-70y | Breast cancer  patients | 0.0 | Validated FFQ | Soy isoflavone | Breast cancer  mortality  (405) | Vital Statistical Registry | Age at diagnosis, education, race-ethnicity, smoking, BMI, physical activity, ER/PR status, TNM stage, chemotherapy, radiotherapy, hormonal therapy, parity, menopausal status, intake of cruciferous vegetable, study | 8 |
| Ollberding et al, 2012, United States^38^ | Multiethnic Cohort Study | 1993-2007  (13.6) | 46,027 women  aged  45-75y | General population | 0.0 | Over-180 item  validated  FFQ | Total soy, tofu,  total soy isoflavones,  daidzein, genistein,  glycitein | Endometrial cancer  incidence  (489) | Cancer registries,  death certificates,  National Death Index | Age, age at cohort entry, race, smoking, BMI, total energy intake, hypertension, diabetes, age at menarche, age at menopause, duration and type of hormone therapy use, duration of oral contraceptive use, parity | 8 |
| Yang et al, 2012, China^15^ | Shanghai Women’s Health Study | 1997-2008  (9.1) | 71,550 women aged  40-70y | General population | 0.0 | 77-item validated FFQ | Soy food,  soy isoflavone | Lung cancer  incidence  (370) | Cancer registries, death certificates | Age, birth year, education, smoking, alcohol, BMI, total energy intake, physical activity, family history of lung cancer, menopausal status, intake of non-soy vegetable, fruit, red meat, non-soy calcium | 9 |
| Hedelin et al, 2011, Sweden^39^ | Women’s Lifestyle and Health  Study | 1991-2007  (16.0) | 47,140 women aged  30-49y | General population | 0.0 | 80-item validated FFQ | Soy isoflavone | Ovarian cancer incidence  (163) | Cancer registries | Age, alcohol, total energy intake, oral contraceptives, age at menarche, parity, HRT use, intake of saturated fat, meat, fish | 9 |
| Butler et al, 2010, Singapore^40^ | Singapore Chinese  Health Study | 1993-2005  (10.7) | 34,028  women  aged  45-74y | General population | 0.0 | 165-item validated FFQ | Soy foods,  soy isoflavone | Breast cancer incidence  (629) | Cancer registries | Age at interview, interview year, education, dialect group, BMI, total energy intake, ﬁrst-degree relative with diagnosis of breast cancer, parity | 8 |
| Shimazu et al, 2010, Japan^70^ | Japan Public Health Center Study | 1995-2005  (8.8) | 76,661 men and women  aged  45-74y | General population | 47.2 | 138-item validated FFQ | Soy isoflavone | Lung cancer  incidence  (659) | Cancer registries,  death certificates | Age, study area, smoking, alcohol, menopausal status, intake of vegetable, fruit, fish | 9 |
| Seow et al, 2009, Singapore^41^ | Singapore Chinese  Health Study | 1993-2005  (9.6) | 35,298  women  aged  45-74y | General population | 0.0 | 165-item validated FFQ | Tofu products and soybean drink,  soy isoflavone | Lung cancer  incidence (298) | Cancer registries,  registry of births and deaths | Age at interview, year at interview, education, dialect group, smoking, cigarettes per day, number of years since quitting, BMI, intake of vegetable, fruit/juice, β-cryptoxanthin, total isothiocyanates | 9 |
| Wang et al, 2009, United  State^10^ | Women’s Health Study | 1992-2007  (11.5) | 38,408 women  aged  ≥45y | General population | 0.0 | 131-item validated FFQ | Tofu | Total cancer  incidence  (3,234) | Medical records, National Death Index,  death certificates | Age, race, smoking, alcohol, BMI, total energy intake, physical activity, family history of colorectal cancer, ovary cancer, breast cancer, randomized treatment assignment use, postmenopausal status, HRT use, multivitamin use, intake of vegetable, fruit, fiber, folate, saturated fat | 7 |
| Yang et al, 2009, China^42^ | Shanghai Women’s Health Study | 1997-2005  (6.4) | 68,412 women  aged  40-70y | General population | 0.0 | 77-item validated FFQ | Soy foods,  soy isoflavone,  soy protein | Colorectal cancer  incidence (321) | Cancer registries,  death certiﬁcates | Age, birth year, education, income, BMI, total energy intake, physical activity, family history of colorectal cancer, menopausal status, intake of vegetable, fruit, red meat, non-soy calcium, non-soy fiber, non-soy folic acid | 9 |
| Akhter et al, 2008, Japan^69^ | Japan Public Health Center Study | 1995-2004  (7.6) | 83,063 men and women  aged  45-74y | General population | 47.0 | 138-item validated FFQ | Soy foods,  miso soup,  soy isoflavone | Colorectal cancer  incidence (886) | Cancer registries,  death certiﬁcates | Age, public health center area, smoking, alcohol, BMI, physical activity, history of diabetes mellitus, menopausal status, current use of female hormones for women, intake of vegetable, fruit, vitamin D, dairy products, fish, meat | 9 |
| Butler et al, 2008, Singapore^43^ | Singapore Chinese  Health Study | 1993-2005  (9.8) | 61,321 men and women  aged  45-74y | General population | NA | 165-item validated FFQ | Soy food,  soy isoflavone | Colorectal cancer  incidence  (961) | Cancer registries,  registry of births and death | Age at interview, sex, education, dialect group, interview year, smoking, alcohol, BMI, total energy intake, physical activity, diabetes at baseline, first-degree relative diagnosed with colorectal cancer | 9 |
| Cutler et al, 2008, United  States^44^ | Iowa Women's Health Study | 1986-2004  (15.2) | 34,708 women  aged  55-69y | General population | 0.0 | 127-item validated FFQ | Soy isoflavone | Total cancer  incidence  (7,534),  lung cancer incidence  (760) | Death records, National Death Index | Age, race, education, smoking, BMI, total energy intake, physical activity, multivitamin use, pack years | 8 |
| Hedelin et al, 2008,  Sweden^45^ | Scandinavian Women’s Lifestyle and Health  Cohort Study | 1991-2004  (13.0) | 45,448 women  aged  30-49y | General population | 0.0 | 80-item validated FFQ | Soy isoflavone | Breast cancer incidence  (1,014) | Nationwide health register, national cancer registries | Age, alcohol, BMI, total energy intake, cancer in sisters or mothers, oral contraceptives, age at first pregnancy, age at menarche, parity, intake of saturated fat | 8 |
| Park et al, 2008, United  State^46^ | Multiethnic Cohort Study | 1993-2003  (8.0) | 82,483  women  aged  45-75y | General population | 100.0 | Over-180 item  validated  FFQ | Soy products,  soy isoflavone, genistein,  daidzein,  glycitein | Prostate cancer  incidence  (4,404) | Cancer registries,  death certificates,  National Death Index | Time since cohort entry, race, education, smoking, BMI, total energy intake, family history of prostate cancer | 7 |
| Travis et al, 2008, United Kingdom^47^ | European Prospective Investigation into Cancer and Nutrition -Oxford | 1993-2003  (7.4) | 37,643 women  aged  20-89y | General population | 0.0 | 178-item  validated FFQ | Soy isoflavone | Breast cancer incidence  (585) | National health service central register | Alcohol, BMI, height, total energy intake, age at menarche, age at first birth and parity, age at first birth, menopausal status, current HRT use | 8 |
| Chang et al, 2007, United  States^48^ | California  Teachers Study | 1995-2003  (8.1) | 97,275 women  aged  50y (median) | General population | 0.0 | Validated FFQ | Tofu/bean curd, total soy isoflavones,  genistein, daidzein | Ovarian cancer  incidence  (280) | Cancer registries,  death file  reports from relatives | Age, race, alcohol, total energy intake, physical activity, parity, oral contraceptive use, menopausal status, hormone therapy use | 7 |
| Fink et al, 2007, United  States^96^ | Long Island Breast Cancer  Study | 1996-2002  (6.0)  max | 1,210 women  aged  25-98y | Breast cancer  patients | 0.0 | Validated FFQ | Total soy isoflavone | Breast cancer  mortality  (113) | National Death Index | Age, total energy intake | 5 |
| Iso et al, 2007, Japan^79^ | Japan Collaborative Cohort Study | 1988-2003  (12.9) | 101,190 men and women  aged  40-79y | General population | 42.2 | 33-item validated FFQ | Miso soup | Total cancer mortality  (5,775) | Death certificates | Age, area | 6 |
| Kurahashi et al,2007, Japan^68^ | Japan Public Health Center Study | 1995-2004  (7.5) | 43,509  men aged  45-74y | General population | 100.0 | 138-item validated FFQ | Soy foods, miso soup, genistein,  daidzein | Prostate cancer  incidence  (307) | Cancer registries,  death certificates | Age, area, smoking, alcohol, BMI, total energy intake, marital status, intake of vegetable, fruit, total fatty acids, dairy | 9 |
| Krish et al, 2007, United  State^49^ | Prostate, Lung, Colorectal and Ovarian Cancer  Screening Trial | 1993-2001  (4.2) | 29,361 men aged  55-74y | General population | 100.0 | 137-item FFQ | Tofu or soybeans | Prostate cancer  incidence  (1,338) | Medical and pathology records,  death certiﬁcates | Age, race, study center, smoking, BMI, total energy intake, physical activity, family, history of prostate cancer, number of prostate cancer screening examinations, diabetes, aspirin, supplemental vitamin E, intake of fat and red meat | 8 |
| Nishio et al, 2007, Japan^50^ | Japan Collaborative Cohort Study | 1988-1997  (7.6) | 30,454  women  aged  40-79y | General population | 0.0 | 33-item validated FFQ | Tofu,  miso soup,  boiled beans | Breast cancer incidence  (145) | Cancer registries | Age, study area, smoking, BMI, total energy intake, physical activity, family history of breast cancer, age at menopause, age at ﬁrst birth, parity, use of exogenous female hormone, intake of vegetable | 9 |
| Oba et al, 2007, Japan^51^ | Takayama Study | 1992-2000  (7.8) | 30,221 men and women  aged  ≥35y | General population | 46.0 | 169-item validated FFQ | Soy products,  soy isoflavone | Colon cancer  incidence  (213) | Cancer registries | Age, smoking, alcohol, BMI, height, physical activity, HRT use (women only), coffee intake | 9 |
| Sakauchi et al, 2007, Japan^80^ | Japan Collaborative Cohort Study | 1988-2003  (13.3) | 64,327 women  aged  40-79y | General population | 0.0 | 33-item validated FFQ | Soybean curd/ tofu | Ovarian cancer  mortality  (77) | Death certificates | Age, education, BMI, physical activity, menopausal status, number of pregnancies, history of sex hormone use | 8 |
| Kurosawa et al, 2006, Japan^81^ | Higashi-yamanashi cohort study | 1989-1999  (11.0) | 8,035  men and women  aged  ≥30y | General population | 45.0 | 29-item FFQ | Bean and bean products | Gastric cancer  mortality  (76) | Death certificates | Age, sex, smoking, intake of vegetable, fruit and highly salted food, mountain herbs | 8 |
| Touillaud et al, 2006, France^52^ | Etude Epidémiologique auprès des femmes de l'Education Nationale  Cohort Study | 1990-2002  (4.2) | 26,868 women  aged  40-65y | General population | 0.0 | 208-item validated FFQ | Total  soy isoflavones | Breast cancer incidence  (402) | NA | Age, area, education, alcohol, BMI, height, total energy intake, family history of breast cancer, age at menarche, personal history of benign breast disease, lifetime use of oral contraceptive, age at first full-term pregnancy, parity | 6 |
| Sauvaget et al, 2005, Japan^53^ | Life Span Study | 1980-1999  (12.5) | 38,576 men and women  aged  34-98y | General population | 38.6 | 22-item validated FFQ | Tofu, miso soup | Gastric cancer  incidence  (1270) | Cancer registries | Age, sex, city, education, smoking, radiation dose | 6 |
| Tokui et al, 2005, Japan^82^ | Japan Collaborative Cohort Study | 1988-1999  (11.0)  max | 110,792 men and women  aged  40-79y | General population | NA | 33-item validated FFQ | Bean curd, miso soup | Gastric cancer  mortality  (859) | Death certificates | Age, sex | 7 |
| Allen et al, 2004, Japan^54^ | Life Span Study | 1963-1996  (16.9) | 18,115 men aged  18-99y | General population | 100.0 | 22-item validated FFQ | Total soya,  tofu, miso soup | Prostate cancer  incidence  (196) | Cancer registries | Age, city of residence, education, calendar period, radiation dose | 7 |
| Keinan-Boker et al, 2004, Netherlands^55^ | European Prospective Investigation into Cancer and Nutrition -Dutch | 1993-2001  (5.2) | 15,555 women  aged  49-70y | General population | 0.0 | 178-item  validated FFQ | Soy isoflavone | Breast cancer incidence  (280) | Municipal registries | Age, education, height, weight, total energy intake, marital status, physical activity, age at first full-term delivery, parity, oral contraceptives or HRT use | 9 |
| Khan et al, 2004, Japan^83^ | Hokkaido cohort | 1984-2002  (14.8 for women;13.8for men) | 3,158  men and women  aged  ≥40y | General population | 48.3 | NA | Soybean curd/ tofu, miso soup | Total cancer mortality  (244) | Vital statistics | Age, smoking, health status, health education, health screening | 7 |
| Kurozawa, et al, 2004, Japan^84^ | Japan Collaborative Cohort Study | 1988-1999  (11.0)  max | 110,792 men and women  aged  40-79y | General population | 41.9 | 33-item validated FFQ | Tofu, miso soup | Hepatocellular  carcinoma  mortality  (401) | Death certificates | Age, sex, history of liver diseases | 7 |
| Nomura et al, 2004, United  State^56^ | NA | 1971-1995  (19.4) | 5,826  men aged  NA | General population | 100.0 | FFQ | Tofu | Prostate cancer  incidence  (304) | Cancer registries | Age, smoking, alcohol, BMI, total energy intake, arm muscle area | 8 |
| Sun et al, 2004, China^57^ | Shanghai Cohort  Study | 1986-2002  (12.8) | 18,224 men aged  45-64y | General population | 100.0 | 45-item  validated FFQ | Total soy,  soy isoflavone,  soy protein | Bladder cancer incidence  (61) | Cancer registries,  death certificates | Age, education, smoking | 8 |
| Ngoan et al, 2002, Japan^85^ | Miyako study | 1986-1999  (10.5) | 13,250 men and women  aged  ＞15y | General population | 44.5 | 254-item FFQ | Tofu, soy milk miso soup | Gastric cancer mortality  (116) | Questionnaire | Age, sex, occupation, smoking, alcohol, history of chronic gastric symptoms, coffee intake | 8 |
| Nagata et al, 2002, Japan^86^ | Takayama study | 1992-1999  (6.9) | 29,079 men and women  aged  ≥35 | General population | 45.9 | 169-item validated FFQ | Soy products | Total cancer mortality  (653) | National Vital Statistics | Age, smoking, alcohol, BMI, total energy intake, marital status, number of children, history of diabetes mellitus | 9 |
| Nagata et al, 2002, Japan^87^ | Takayama study | 1992-1999  (6.9) | 30,304 men and women  aged  ≥35y | General population | 45.8 | 169-item validated FFQ | Soy products, non-fermented  fermented | Gastric cancer mortality  (121) | National Vital Statistics | Age, smoking, BMI at age about 21 years, total energy intake, marital status, age at menarche, coffee intake, intake of salt and rice | 9 |
| Horn-Ross et al, 2002, United States^58^ | California Teachers Study | 1995-1998  (2.0) | 111,526 women  aged  21-103y | General population | 0.0 | 103-item validated FFQ | Soy isoflavone | Breast cancer incidence  (711) | Cancer registries | Age, race, total energy intake, physical activity, family history of breast cancer, age at menarche, nulliparity/age at first full-term pregnancy, an interaction term for BMI and menopausal status | 7 |
| Sun et al, 2002, Singapore^59^ | Singapore Chinese  Health Study | 1993-2000  (5.2) | 63,257 men and women  aged  45-74y | General population | 55.8 | 165-item validated FFQ | Total soy, soy isoflavone,  soy protein | Bladder cancer incidence  (61) | Cancer registries, registry of Births and Deaths | Age, sex, year of recruitment, dialect group, education, smoking | 7 |
| Ozasa et al,  2001, Japan^88^ | Japan Collaborative Cohort Study | 1988-1997  (7.6) | 98,248 men and women  aged  40-79 y | General population | 54.3 | 33-item validated FFQ | Tofu, miso soup | Lung cancer mortality  (572) | Death certificates | Age, smoking, smoking index, time since quitting smoking, parents’ history of lung cancer | 8 |
| Key et al, 1999, Japan^60^ | Life Span Study | 1969-1993  1979-1993  (14.1) | 34,759 women  aged  NA | General population | 0.0 | FFQ | Tofu, miso soup | Breast cancer incidence  (427) | Cancer registries,  Japanese family registration system | Age, calendar period, city, age at time of bombing, radiation dose | 6 |
| Galanis et al, 1998, United  States^61^ | NA | 1975-1994  (14.8) | 11,907 men and women  aged  ≥18y | General population | 47.1 | 19-item FFQ | Miso soup | Gastric cancer  incidence  (108) | Cancer registries | Age, sex, smoking, alcohol education, Japanese place of birth | 8 |
| Jacobsen et al, 1998, United  States^62^ | Adventist Health Study | 1976-1982  (6.0)  (max) | 12,395 men aged  ≥25y | General population | 100.0 | 200-item FFQ | Soy milk | Prostate cancer  incidence  (225) | Medical records,  cancer registries | Age, BMI, age at first marriage coffee intake, intake of fruit, whole fat milk, eggs and citrus | 6 |
| Inoue et al, 1996, Japan^63^ | NA | 1985-1995  (6.0) | 5,373  men and women  aged  NA | Patients  who undergo gastroscopy | 52.5 | FFQ | Soybean-paste soup | Gastric cancer  incidence  (69) | Medical record, cancer registries,  death certificates | Age, sex | 6 |
| Kato et al,  1992, Japan^89^ | NA | 1985-1991  (5.7) | 9,753  men and women  aged  ≥30y | General population | NA | FFQ | Miso soup | Gastric cancer mortality  (57) | Death certificates | Age, sex | 8 |
| Nomura et al, 1990, United  States^64^ | Japan-Hawaii Cancer Study | 1965-1986  (19.0) | 7,990  men aged  46-65y | General population | 100.0 | FFQ | Tofu, miso soup | Gastric cancer incidence  (150) | Cancer registries | Age | 7 |
| Severson et al, 1989, United  States^65^ | Japan-Hawaii Cancer Study | 1965-1986  (17.5) | 7,999  men aged  46-65y | General population | 100.0 | FFQ | Tofu, miso soup | Prostate cancer  incidence  (174) | Cancer registries | Age | 7 |

**Table S2 ǀ** Newcastle-Ottawa scale assessments for cohort studies on soy, soy isoflavones, soy protein and cancer incidence and mortality

| **First author, year** | **Selection** | | | | Comparability | Assessment of exposure | | | **Total score** |
| --- | --- | --- | --- | --- | --- | --- | --- | --- | --- |
|  | Representativ  eness | Selection of the  non-exposed cohort | Exposure  assessment | Demonstration of  outcome not  present at start | Comparability of  cohorts on the basis of  the design or analysis | Outcome ascertainment | Adequate follow-up time  (>5 years) | Adequacy  of follow up  (>80%) |  |
| Shirabe, 2021 | 1 | 1 | 1 | 1 | 2 | 1 | 1 | 1 | 9 |
| Nozue, 2021 | 1 | 1 | 1 | 1 | 2 | 1 | 1 | 1 | 9 |
| Abe, 2021 | 1 | 1 | 1 | 1 | 2 | 1 | 1 | 1 | 9 |
| Ho, 2021 | 0 | 1 | 1 | 1 | 2 | 1 | 1 | 1 | 8 |
| Wei, 2020 | 1 | 1 | 1 | 1 | 2 | 1 | 1 | 1 | 9 |
| Fraser, 2020 | 0 | 1 | 1 | 1 | 2 | 1 | 1 | 0 | 7 |
| Yamagiwa, 2020 | 1 | 1 | 1 | 1 | 2 | 1 | 1 | 1 | 9 |
| Katagiri, 2020 | 1 | 1 | 1 | 1 | 2 | 1 | 1 | 1 | 9 |
| Sawada, 2020 | 1 | 1 | 1 | 1 | 2 | 1 | 1 | 1 | 9 |
| Minami, 2020 | 0 | 1 | 1 | 1 | 2 | 1 | 1 | 1 | 8 |
| Dunneram, 2019 | 1 | 1 | 1 | 1 | 2 | 1 | 1 | 0 | 8 |
| Paul, 2019 | 1 | 1 | 1 | 1 | 2 | 1 | 1 | 0 | 8 |
| Wada, 2018 | 1 | 1 | 1 | 1 | 2 | 1 | 1 | 1 | 9 |
| Reger, 2018 | 1 | 1 | 1 | 1 | 2 | 1 | 1 | 1 | 9 |
| Tang, 2017, SWHS | 1 | 1 | 1 | 1 | 2 | 1 | 1 | 1 | 9 |
| Tang, 2017, SWHS | 1 | 1 | 1 | 1 | 2 | 1 | 1 | 1 | 9 |
| Baglia, 2016 | 1 | 1 | 1 | 1 | 2 | 1 | 1 | 1 | 9 |
| Umesawa, 2016 | 1 | 1 | 1 | 1 | 2 | 1 | 1 | 1 | 9 |
| Hedelin, 2016 | 0 | 1 | 1 | 1 | 2 | 1 | 1 | 1 | 8 |
| Leo, 2016 | 0 | 1 | 1 | 1 | 2 | 1 | 0 | 1 | 7 |
| Wada, 2015 | 1 | 1 | 1 | 1 | 2 | 1 | 1 | 1 | 9 |
| Budhathoki, 2015 | 1 | 1 | 1 | 1 | 2 | 1 | 1 | 1 | 9 |
| Yamasaki, 2015 | 0 | 1 | 1 | 1 | 2 | 1 | 1 | 1 | 8 |
| Kyrø, 2015 | 0 | 1 | 1 | 1 | 2 | 1 | 1 | 0 | 7 |
| Morimoto, 2014 | 1 | 1 | 1 | 1 | 2 | 1 | 1 | 0 | 8 |
| Wang, 2014 | 1 | 1 | 1 | 1 | 2 | 1 | 1 | 1 | 9 |
| Ros, 2014 | 1 | 1 | 1 | 1 | 2 | 1 | 1 | 1 | 9 |
| Wada, 2013 | 1 | 1 | 1 | 1 | 2 | 1 | 1 | 1 | 9 |
| Kweon, 2013, SWHS | 1 | 1 | 1 | 1 | 2 | 1 | 1 | 1 | 9 |
| Kweon, 2013, SMHS | 1 | 1 | 1 | 1 | 2 | 1 | 1 | 1 | 9 |
| Ko, 2013 | 1 | 1 | 1 | 1 | 2 | 1 | 1 | 0 | 8 |
| Ros, 2013 | 1 | 1 | 1 | 1 | 2 | 1 | 1 | 0 | 8 |
| Conroy, 2013 | 0 | 1 | 1 | 1 | 2 | 1 | 1 | 1 | 8 |
| Hara, 2012 | 1 | 1 | 1 | 1 | 2 | 1 | 1 | 1 | 9 |
| Ollberding, 2012 | 1 | 1 | 1 | 1 | 2 | 1 | 1 | 0 | 8 |
| Yang, 2012 | 1 | 1 | 1 | 1 | 2 | 1 | 1 | 1 | 9 |
| Nechuta, 2012,  LACE&WHEL | 0 | 1 | 1 | 1 | 2 | 1 | 1 | 1 | 8 |
| Nechuta, 2012, SBCSS | 0 | 1 | 1 | 1 | 2 | 1 | 1 | 1 | 8 |
| Hedelin, 2011 | 1 | 1 | 1 | 1 | 2 | 1 | 1 | 1 | 9 |
| Butler, 2010 | 1 | 1 | 1 | 1 | 1 | 1 | 1 | 1 | 8 |
| Shimazu, 2010 | 1 | 1 | 1 | 1 | 2 | 1 | 1 | 1 | 9 |
| Yang, 2009 | 1 | 1 | 1 | 1 | 2 | 1 | 1 | 1 | 9 |
| Seow, 2009 | 1 | 1 | 1 | 1 | 2 | 1 | 1 | 1 | 9 |
| Wang, 2009 | 0 | 1 | 1 | 1 | 2 | 1 | 1 | 0 | 7 |
| Butler, 2008 | 1 | 1 | 1 | 1 | 2 | 1 | 1 | 1 | 9 |
| Akhter, 2008 | 1 | 1 | 1 | 1 | 2 | 1 | 1 | 1 | 9 |
| Park, 2008 | 1 | 1 | 1 | 1 | 1 | 1 | 1 | 0 | 7 |
| Cutler, 2008 | 0 | 1 | 1 | 1 | 2 | 1 | 1 | 1 | 8 |
| Hedelin, 2008 | 0 | 1 | 1 | 1 | 2 | 1 | 1 | 1 | 8 |
| Travis, 2008 | 1 | 1 | 1 | 1 | 1 | 1 | 1 | 1 | 8 |
| Kurahashi, 2007 | 1 | 1 | 1 | 1 | 2 | 1 | 1 | 1 | 9 |
| Chang, 2007 | 0 | 1 | 1 | 1 | 1 | 1 | 1 | 1 | 7 |
| Nishio, 2007 | 1 | 1 | 1 | 1 | 2 | 1 | 1 | 1 | 9 |
| Krish, 2007 | 1 | 1 | 1 | 1 | 2 | 1 | 0 | 1 | 8 |
| Oba, 2007 | 1 | 1 | 1 | 1 | 2 | 1 | 1 | 1 | 9 |
| Iso, 2007 | 0 | 1 | 1 | 1 | 1 | 1 | 1 | 0 | 6 |
| Sakauchi, 2007 | 0 | 1 | 1 | 1 | 2 | 1 | 1 | 1 | 8 |
| Fink, 2007 | 0 | 1 | 1 | 1 | 1 | 1 | 0 | 0 | 5 |
| Touillaud, 2006 | 0 | 1 | 1 | 1 | 2 | 0 | 1 | 0 | 6 |
| Kurosawa, 2006 | 1 | 1 | 1 | 1 | 1 | 1 | 1 | 1 | 8 |
| Sauvaget, 2005 | 0 | 1 | 1 | 1 | 1 | 1 | 1 | 0 | 6 |
| Tokui, 2005 | 1 | 1 | 1 | 1 | 1 | 1 | 1 | 0 | 7 |
| Sun, 2004 | 1 | 1 | 1 | 1 | 1 | 1 | 1 | 1 | 8 |
| Allen, 2004 | 0 | 1 | 1 | 1 | 1 | 1 | 1 | 1 | 7 |
| Nomura, 2004 | 1 | 1 | 1 | 1 | 2 | 1 | 1 | 0 | 8 |
| Boker, 2004 | 1 | 1 | 1 | 1 | 2 | 1 | 1 | 1 | 9 |
| Khan, 2004 | 1 | 1 | 0 | 1 | 1 | 1 | 1 | 1 | 7 |
| Kurozawa, 2004 | 1 | 1 | 1 | 1 | 1 | 1 | 1 | 0 | 7 |
| Sun, 2002 | 1 | 1 | 1 | 1 | 1 | 1 | 1 | 0 | 7 |
| Ross, 2002 | 0 | 1 | 1 | 1 | 2 | 1 | 0 | 1 | 7 |
| Ngoan, 2002 | 1 | 1 | 1 | 1 | 2 | 1 | 1 | 0 | 8 |
| Nagata, 2002 | 1 | 1 | 1 | 1 | 2 | 1 | 1 | 1 | 9 |
| Nagata, 2002 | 1 | 1 | 1 | 1 | 2 | 1 | 1 | 1 | 9 |
| Ozasa, 2001 | 0 | 1 | 1 | 1 | 2 | 1 | 1 | 1 | 8 |
| Key, 1999 | 0 | 1 | 1 | 1 | 1 | 1 | 1 | 0 | 6 |
| Jacobsen, 1998 | 0 | 1 | 1 | 1 | 1 | 1 | 1 | 0 | 6 |
| Galanis, 1998 | 1 | 1 | 1 | 1 | 1 | 1 | 1 | 1 | 8 |
| Inoue, 1996 | 0 | 1 | 1 | 1 | 1 | 1 | 1 | 0 | 6 |
| Kato, 1992 | 1 | 1 | 1 | 1 | 1 | 1 | 1 | 1 | 8 |
| Nomura, 1990 | 1 | 1 | 1 | 1 | 1 | 1 | 1 | 0 | 7 |
| Severson, 1989 | 1 | 1 | 1 | 1 | 1 | 1 | 1 | 0 | 7 |

**Table S3 ǀ** Subgroup analyses of the association between soy and cancer incidence

| **Subgroup analysis** | **n** | **RR (95%CI)** | ***I^2^*** | ***Ph^*^*** | ***Ph^†^*** |
| --- | --- | --- | --- | --- | --- |
| **Overall analysis** | 35 | 0.90 (0.83-0.96) | 57.8 | < 0.001 | NA |
| **Sex** |  |  |  |  | 0.28 |
| Female | 16 | 0.88 (0.83-0.95) | 23.5 | 0.19 |  |
| Male | 8 | 0.86 (0.67-1.10) | 43.2 | 0.09 |  |
| Female and male | 11 | 0.97 (0.83-1.14) | 76.2 | < 0.001 |  |
| **Country of origin** |  |  |  |  | 0.66 |
| United States | 11 | 0.91 (0.84-0.98) | 0.0 | 0.48 |  |
| Asia | 23 | 0.89 (0.80-0.99) | 68.4 | < 0.001 |  |
| Europe | 1 | 0.97 (0.90-1.04) | NA | NA |  |
| **Follow-up** |  |  |  |  | 0.40 |
| < 10 y | 15 | 0.86 (0.76-0.97) | 54.5 | 0.01 |  |
| ≥10y | 20 | 0.93 (0.85-1.01) | 55.5 | 0.001 |  |
| **Adjusted for family history of cancer** |  |  |  |  | 0.78 |
| Yes | 13 | 0.91 (0.82-1.00) | 43.7 | 0.05 |  |
| No | 22 | 0.88 (0.80-0.98) | 63.4 | < 0.001 |  |
| **Type of individual soy foods**^¶^ |  |  |  |  | 0.23 |
| Tofu | 16 | 0.96 (0.86-1.06) | 19.2 | 0.23 |  |
| Soy milk | 6 | 0.96 (0.75-1.24) | 70.0 | 0.01 |  |
| Miso | 11 | 1.06 (0.95-1.17) | 27.9 | 0.18 |  |
| Natto | 1 | 1.06 (0.98-1.13) | NA | NA |  |

CI, confidence interval; NA, not available; RR, relative risk.

*P for heterogeneity within subgroups.

*†*P for heterogeneity between subgroups with a meta-regression analysis.

¶Individual soy food, including tofu, soy milk, miso and natto, were not reported in all 35

studies.

**Table S4 ǀ** Subgroup analyses of the association between soy isoflavones and cancer incidence

| **Subgroup** | **n** | **RR (95%CI)** | ***I^2^*** | ***Ph^*^*** | ***Ph^†^*** |
| --- | --- | --- | --- | --- | --- |
| **Overall analysis** | 33 | 0.94 (0.89-0.99) | 52.6 | < 0.001 | NA |
| **Sex** |  |  |  |  | 0.14 |
| Female | 22 | 0.91 (0.87-0.96) | 7.2 | 0.36 |  |
| Male | 4 | 1.11 (0.95-1.29) | 58.7 | 0.06 |  |
| Female and male | 7 | 0.94 (0.82-1.07) | 67.1 | 0.01 |  |
| **Country of origin** |  |  |  |  | 0.44 |
| United States | 7 | 0.95 (0.86-1.06) | 51.2 | 0.06 |  |
| Asia | 19 | 0.90 (0.82-0.99) | 65.5 | < 0.001 |  |
| Europe | 7 | 0.95 (0.90-1.01) | 0.0 | 0.91 |  |
| **Follow-up** |  |  |  |  | 0.65 |
| < 10 y | 15 | 0.95 (0.88-1.03) | 44.3 | 0.03 |  |
| ≥10y | 18 | 0.92 (0.85-1.00) | 59.9 | 0.001 |  |
| **Adjusted for family history of cancer** |  |  |  |  | 0.47 |
| Yes | 15 | 0.96 (0.90-1.03) | 40.6 | 0.05 |  |
| No | 18 | 0.91 (0.84-0.99) | 59.7 | 0.001 |  |

CI, confidence interval; NA, not available; RR, relative risk.

*P for heterogeneity within subgroups.

†P for heterogeneity between subgroups with a meta-regression analysis.


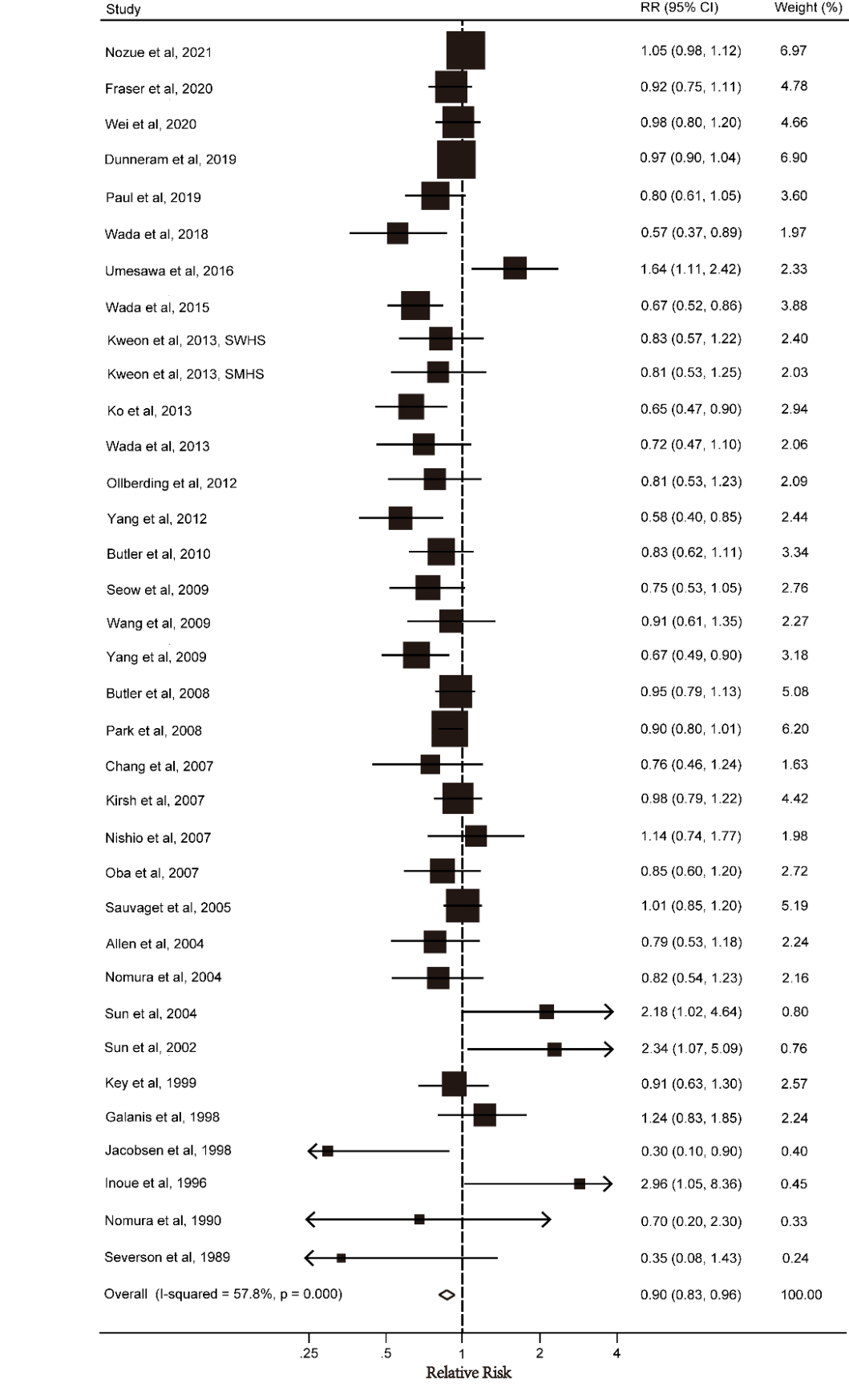


**Figure S1 ǀ** Forest plot for association between soy intake and risk of overall cancer incidence, expressed as comparison between highest and lowest categories of soy intake. The size of the black squares reflects the relative statistical weight of study-specific estimate, horizontal lines indicate 95% CIs. The diamond indicates the pooled RR estimates with 95% CI. CI, confidence interval; RR, relative risk; SWHS, Shanghai Women's Health Study; SMHS, Shanghai Men's Health Study.

**
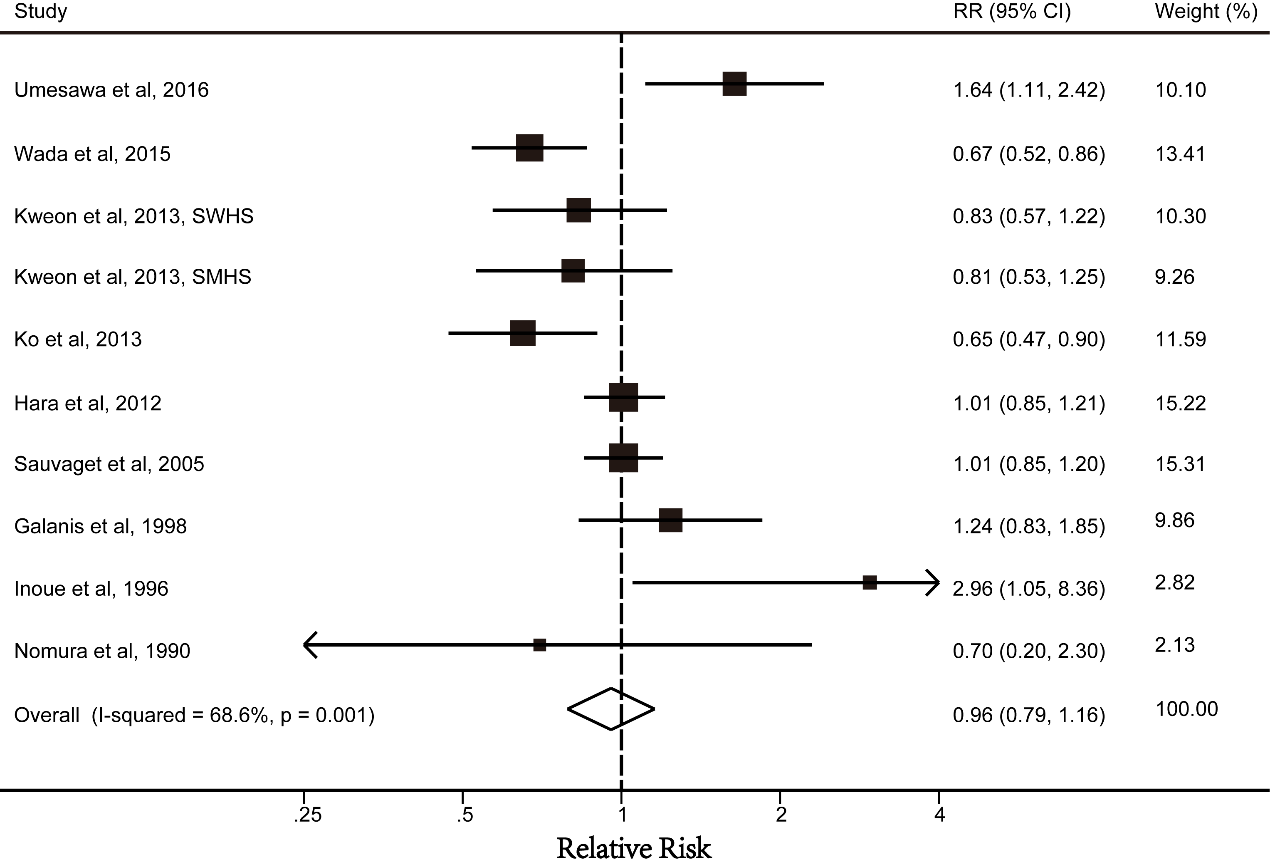
**

**Figure S2 ǀ** Forest plot for association between soy intake and risk of gastric cancer, expressed as comparison between highest and lowest categories of soy intake. The size of the black squares reflects the relative statistical weight of study-specific estimate, horizontal lines indicate 95% CIs. The diamond indicates the pooled RR estimates with 95% CI. CI, confidence interval; RR, relative risk; SWHS, Shanghai Women's Health Study; SMHS, Shanghai Men's Health Study.


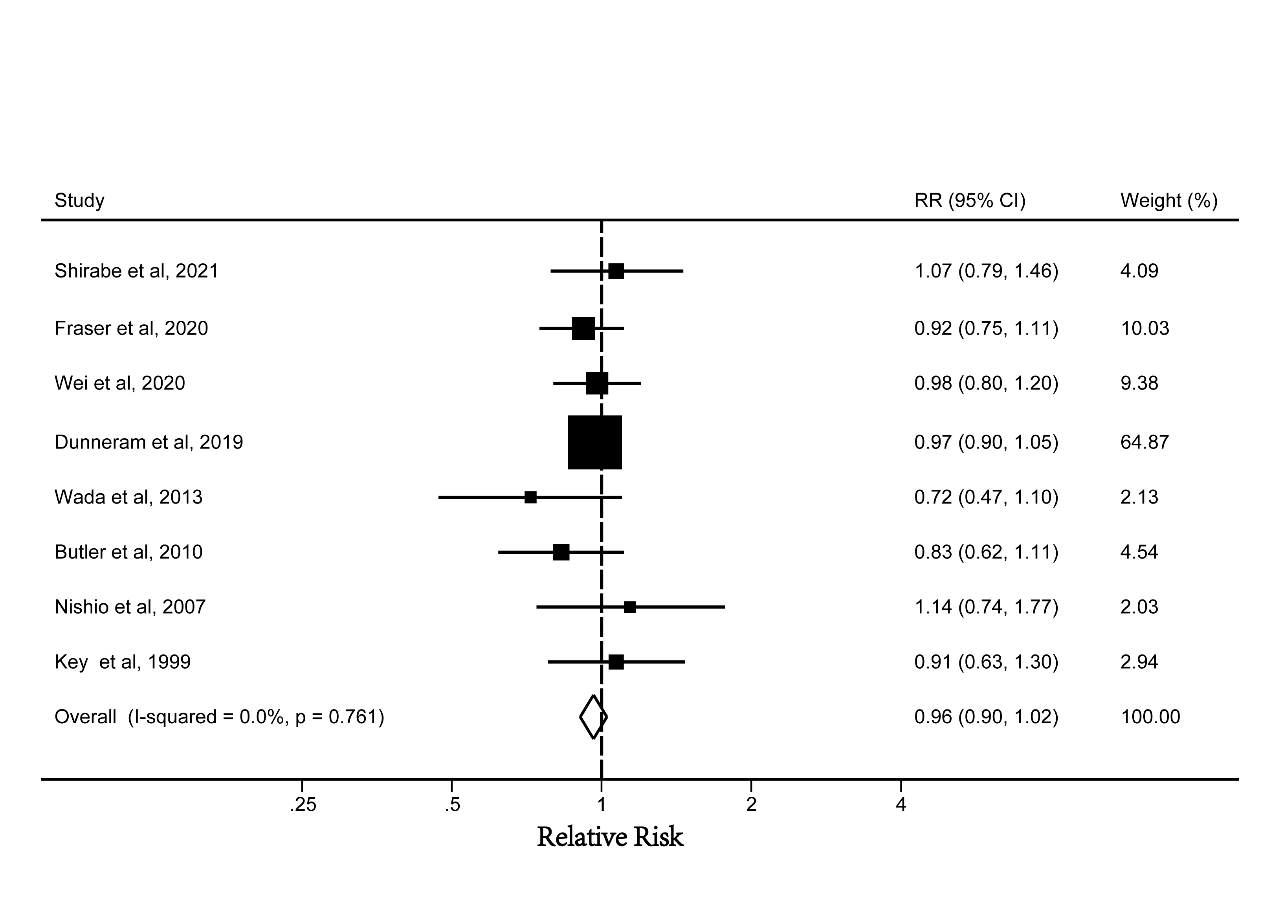


**Figure S3** **ǀ** Forest plot for association between soy intake and risk of breast cancer, expressed as comparison between highest and lowest categories of soy intake. The size of the black squares reflects the relative statistical weight of study-specific estimate, horizontal lines indicate 95% CIs. The diamond indicates the pooled RR estimates with 95% CI. CI, confidence interval; RR, relative risk.

**
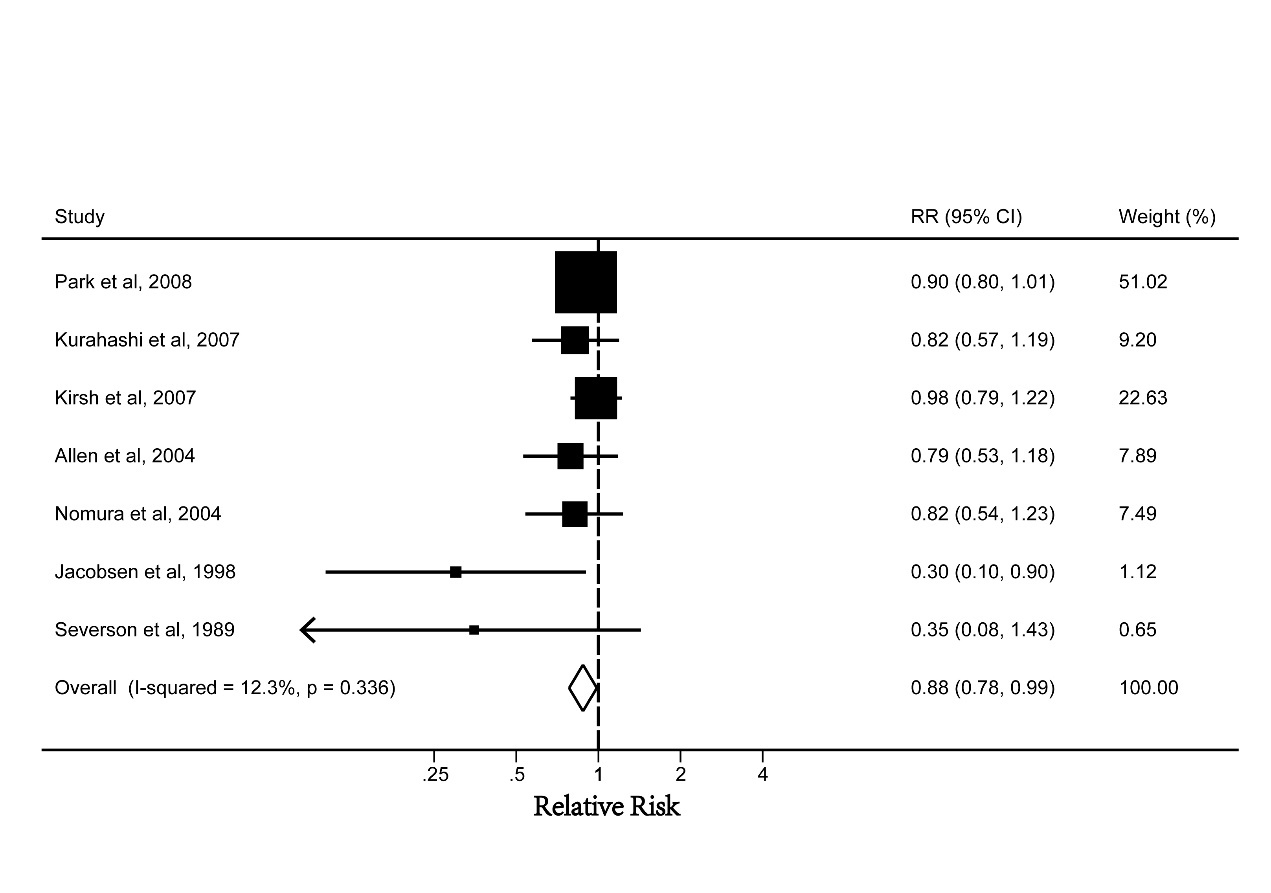
**

**Figure S4 ǀ** Forest plot for association between soy intake and risk of prostate cancer, expressed as comparison between highest and lowest categories of soy intake. The size of the black squares reflects the relative statistical weight of study-specific estimate, horizontal lines indicate 95% CIs. The diamond indicates the pooled RR estimates with 95% CI. CI, confidence interval; RR, relative risk.

**
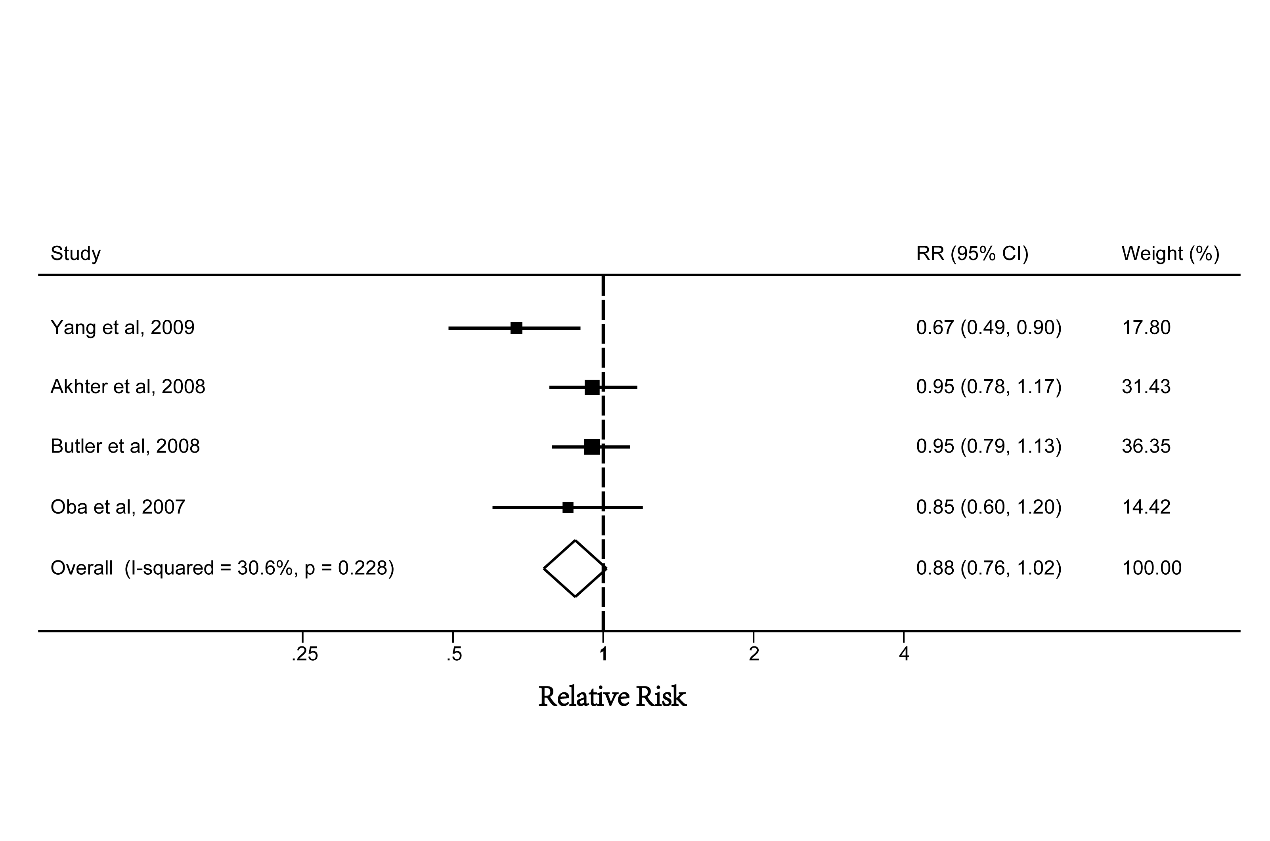
**

**Figure S5 ǀ** Forest plot for association between soy intake and risk of colorectal cancer, expressed as comparison between highest and lowest categories of soy intake. The size of the black squares reflects the relative statistical weight of study-specific estimate, horizontal lines indicate 95% CIs. The diamond indicates the pooled RR estimates with 95% CI. CI, confidence interval; RR, relative risk.

**
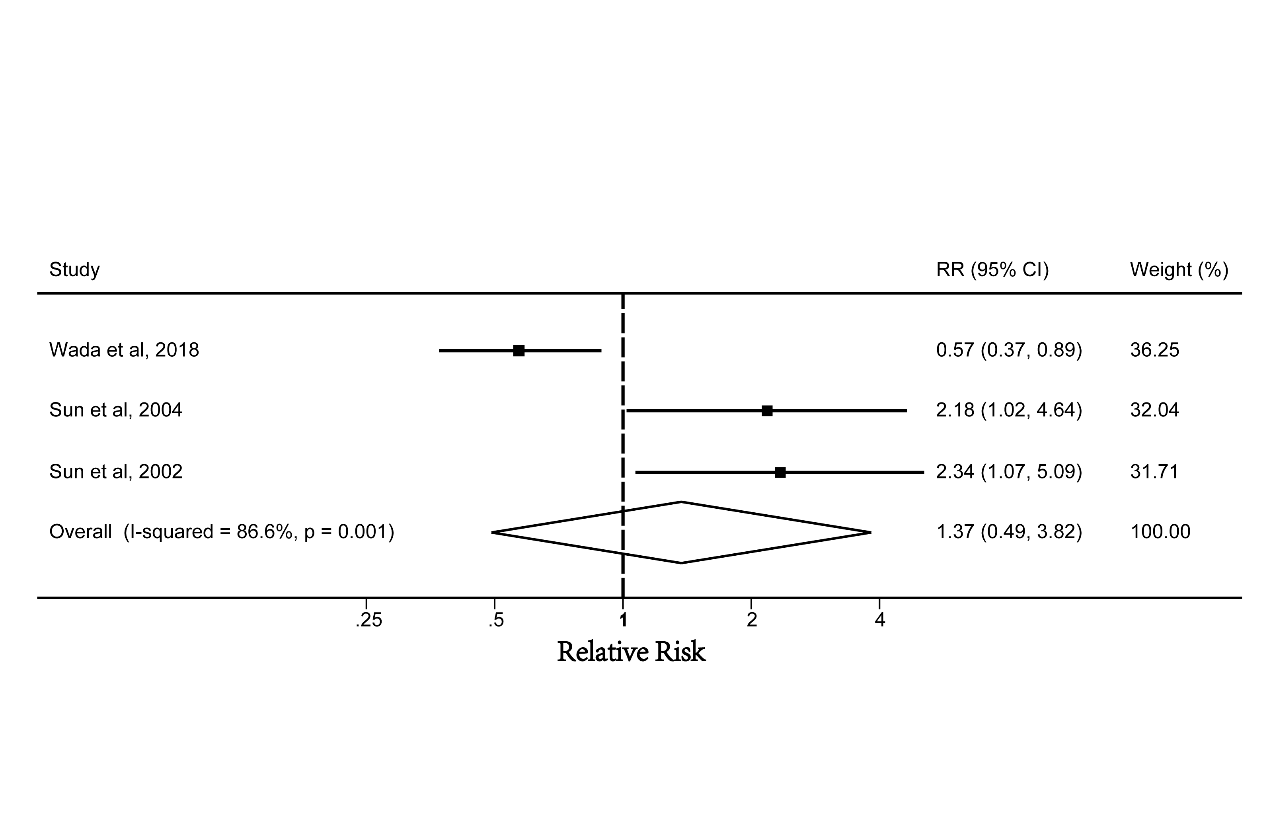
**

**Figure S6 ǀ** Forest plot for association between soy intake and risk of bladder cancer, expressed as comparison between highest and lowest categories of soy intake. The size of the black squares reflects the relative statistical weight of study-specific estimate, horizontal lines indicate 95% CIs. The diamond indicates the pooled RR estimates with 95% CI. CI, confidence interval; RR, relative risk.

**
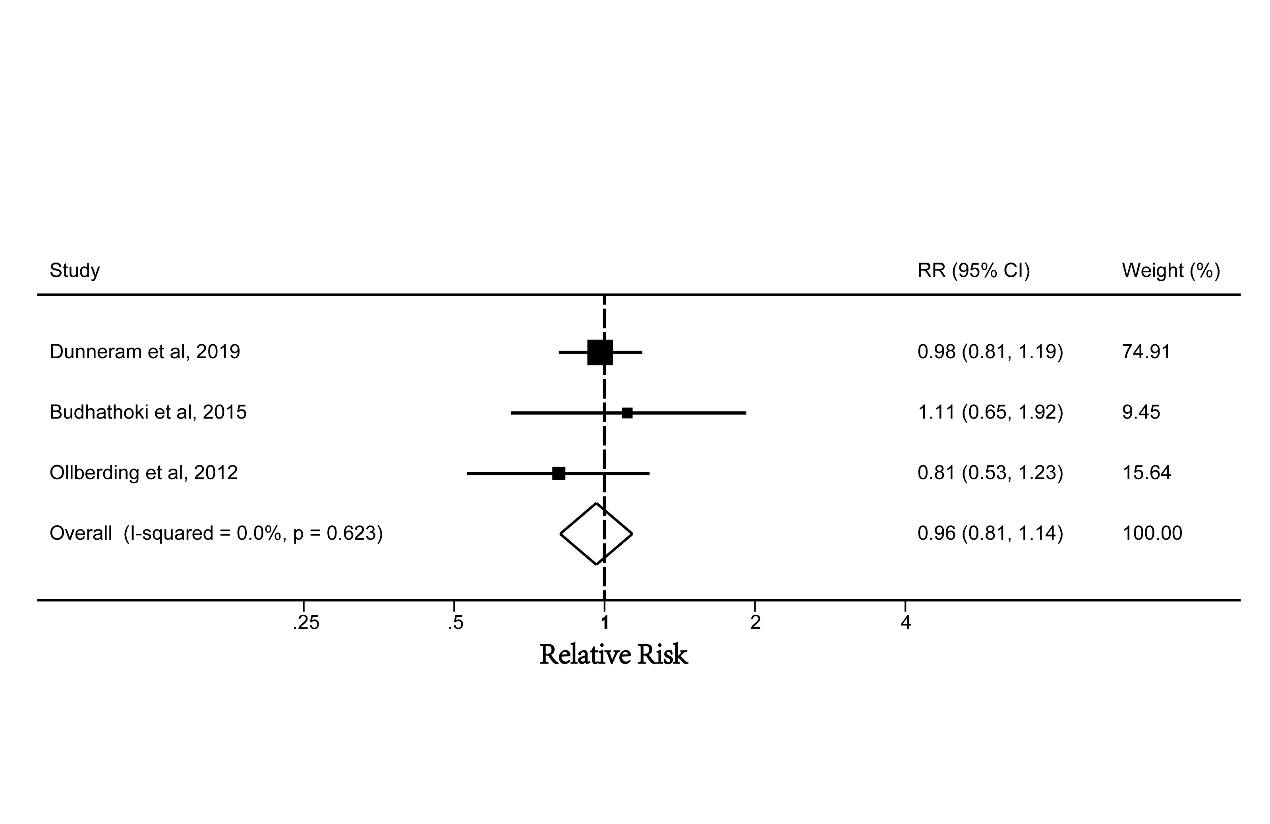
**

**Figure S7 ǀ** Forest plot for association between soy intake and risk of endometrial cancer, expressed as comparison between highest and lowest categories of soy intake. The size of the black squares reflects the relative statistical weight of study-specific estimate, horizontal lines indicate 95% CIs. The diamond indicates the pooled RR estimates with 95% CI. CI, confidence interval; RR, relative risk.

**
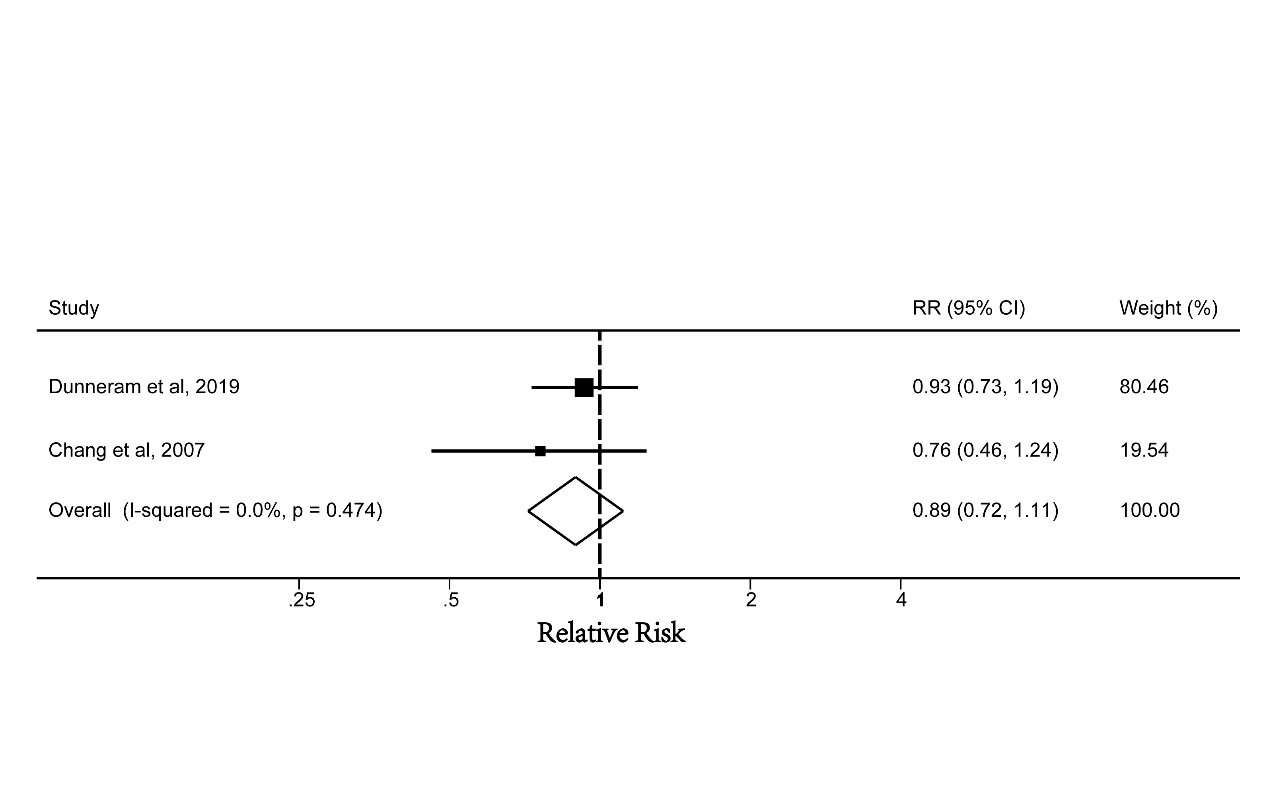
**

**Figure S8 ǀ** Forest plot for association between soy intake and risk of ovarian cancer, expressed as comparison between highest and lowest categories of soy intake. The size of the black squares reflects the relative statistical weight of study-specific estimate, horizontal lines indicate 95% CIs. The diamond indicates the pooled RR estimates with 95% CI. CI, confidence interval; RR, relative risk.

**
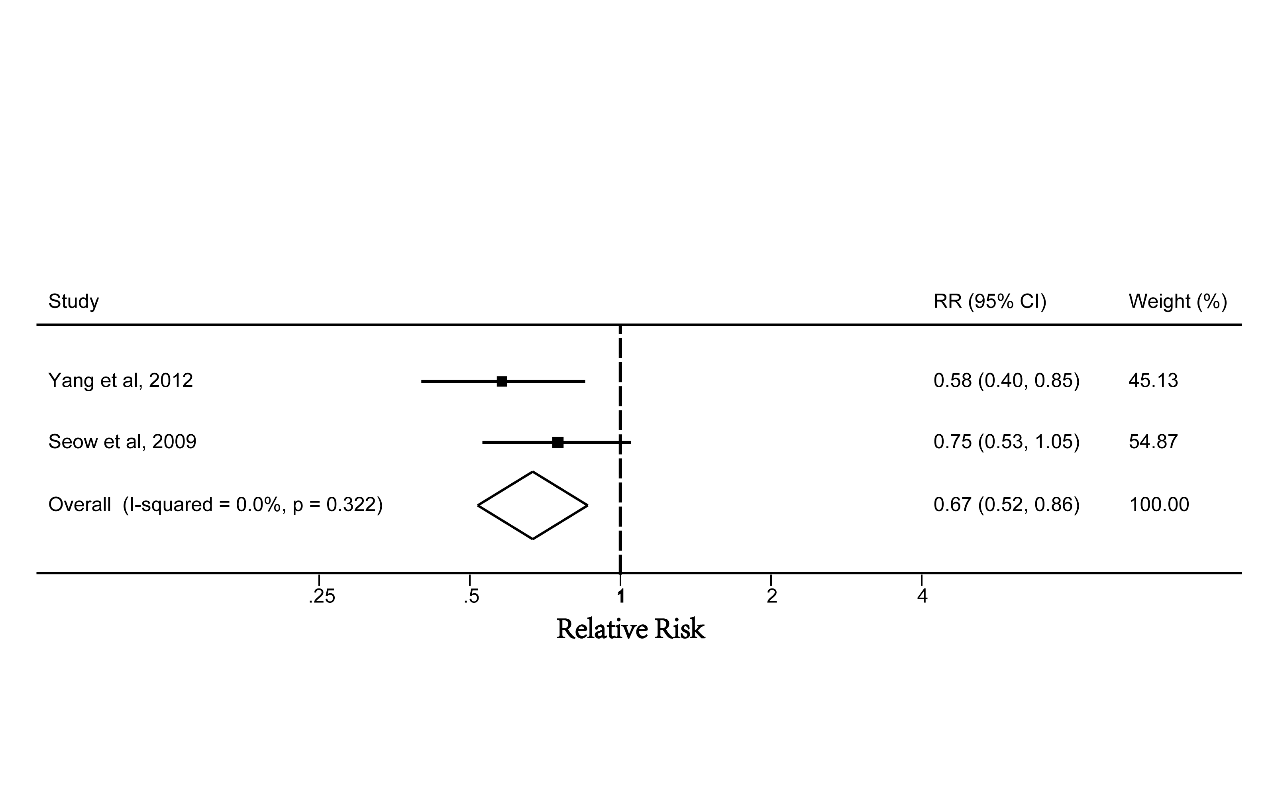
**

**Figure S9 ǀ** Forest plot for association between soy intake and risk of lung cancer, expressed as comparison between highest and lowest categories of soy intake. The size of the black squares reflects the relative statistical weight of study-specific estimate, horizontal lines indicate 95% CIs. The diamond indicates the pooled RR estimates with 95% CI. CI, confidence interval; RR, relative risk.

**
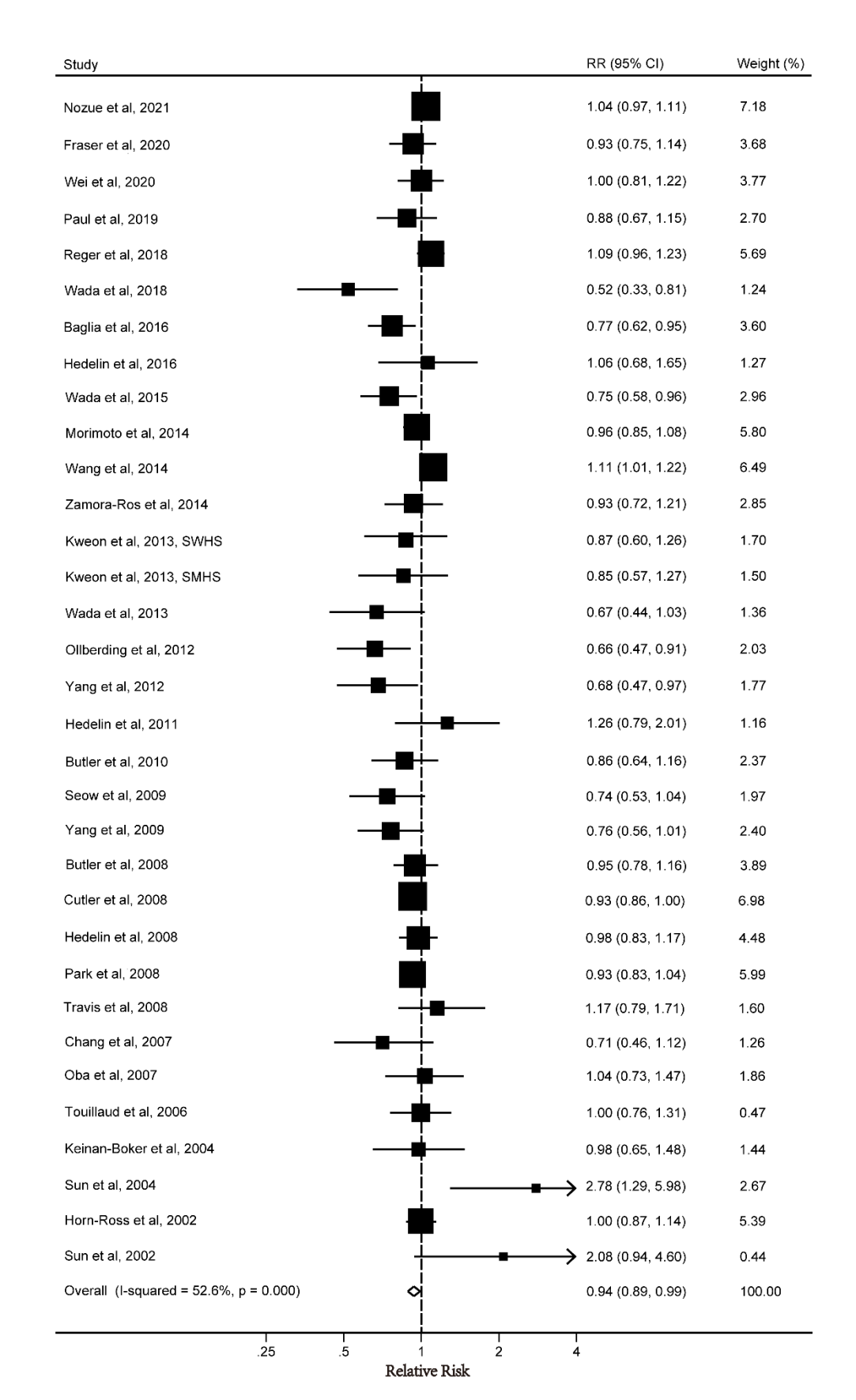
**

**Figure S10 ǀ** Forest plot for association between soy isoflavones intake and risk of overall cancer incidence, expressed as comparison between highest and lowest categories of soy isoflavones intake. The size of the black squares reflects the relative statistical weight of study-specific estimate, horizontal lines indicate 95% CIs. The diamond indicates the pooled RR estimates with 95% CI. CI, confidence interval; RR, relative risk; SWHS, Shanghai Women's Health Study; SMHS, Shanghai Men's Health Study.

**
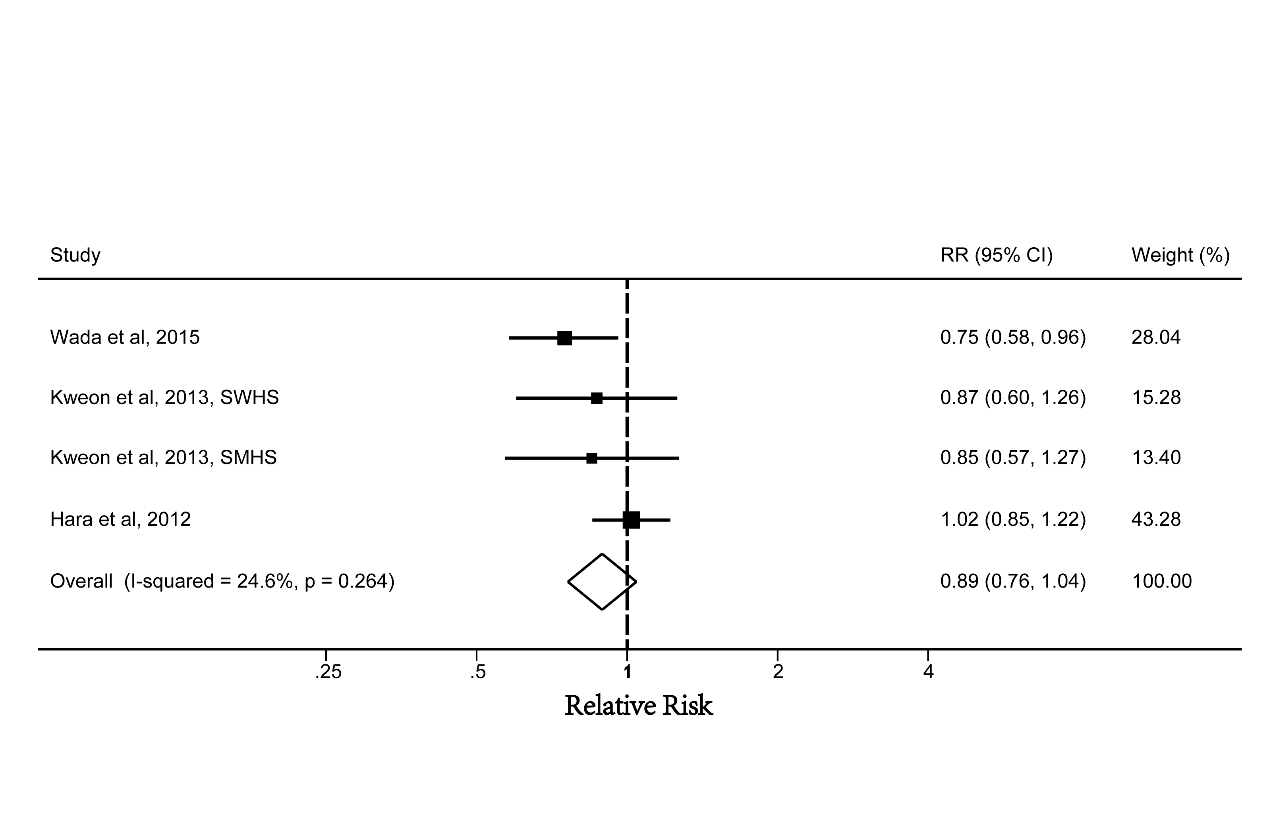
**

**Figure S11 ǀ** Forest plot for association between soy isoflavones intake and risk of gastric cancer, expressed as comparison between highest and lowest categories of soy isoflavones intake. The size of the black squares reflects the relative statistical weight of study-specific estimate, horizontal lines indicate 95% CIs. The diamond indicates the pooled RR estimates with 95% CI. CI, confidence interval; RR, relative risk; SWHS, Shanghai Women's Health Study; SMHS, Shanghai Men's Health Study.

**
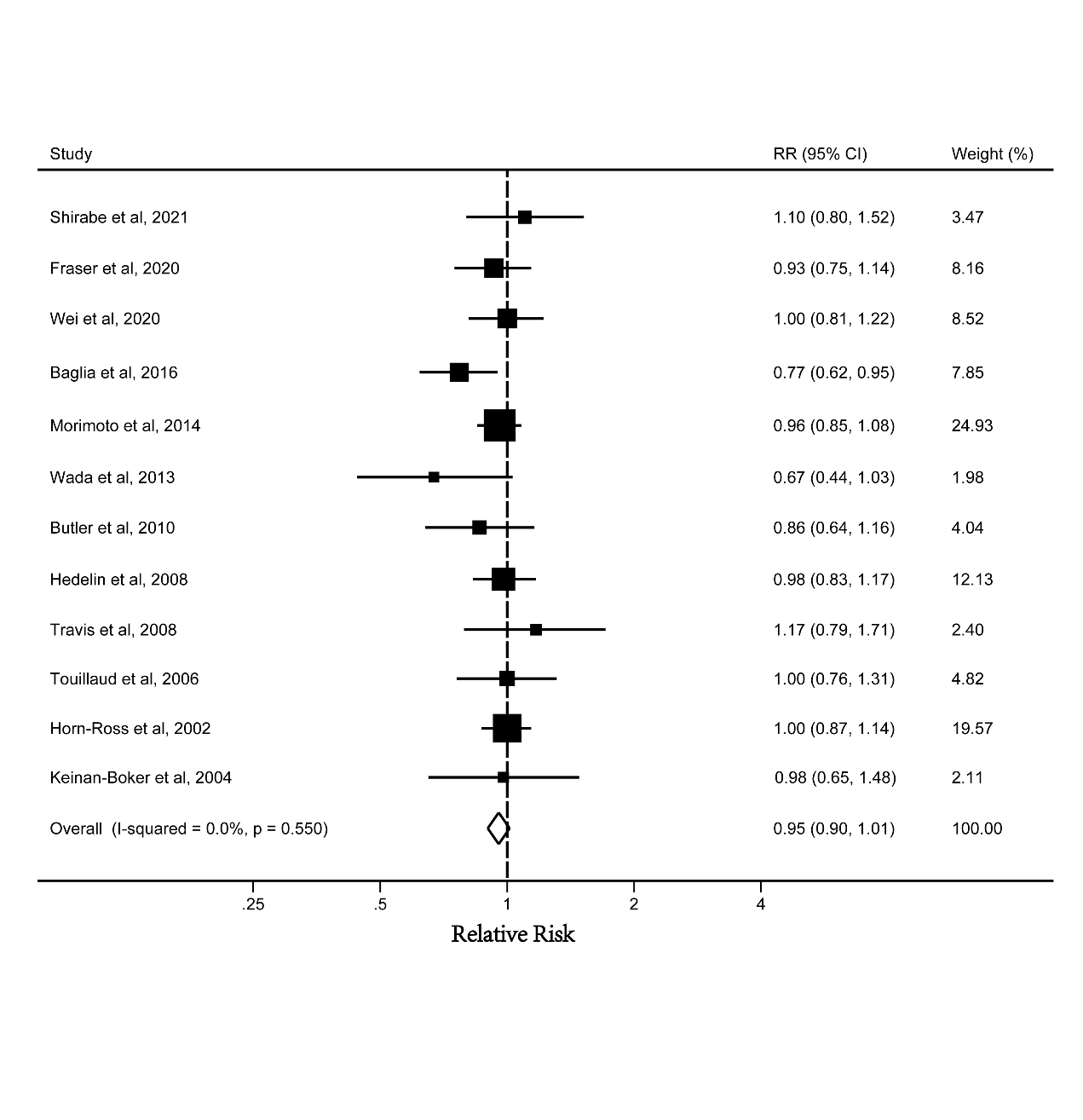
**

**Figure S12 ǀ** Forest plot for association between soy isoflavones intake and risk of breast cancer, expressed as comparison between highest and lowest categories of soy isoflavones intake. The size of the black squares reflects the relative statistical weight of study-specific estimate, horizontal lines indicate 95% CIs. The diamond indicates the pooled RR estimates with 95% CI. CI, confidence interval; RR, relative risk.

**
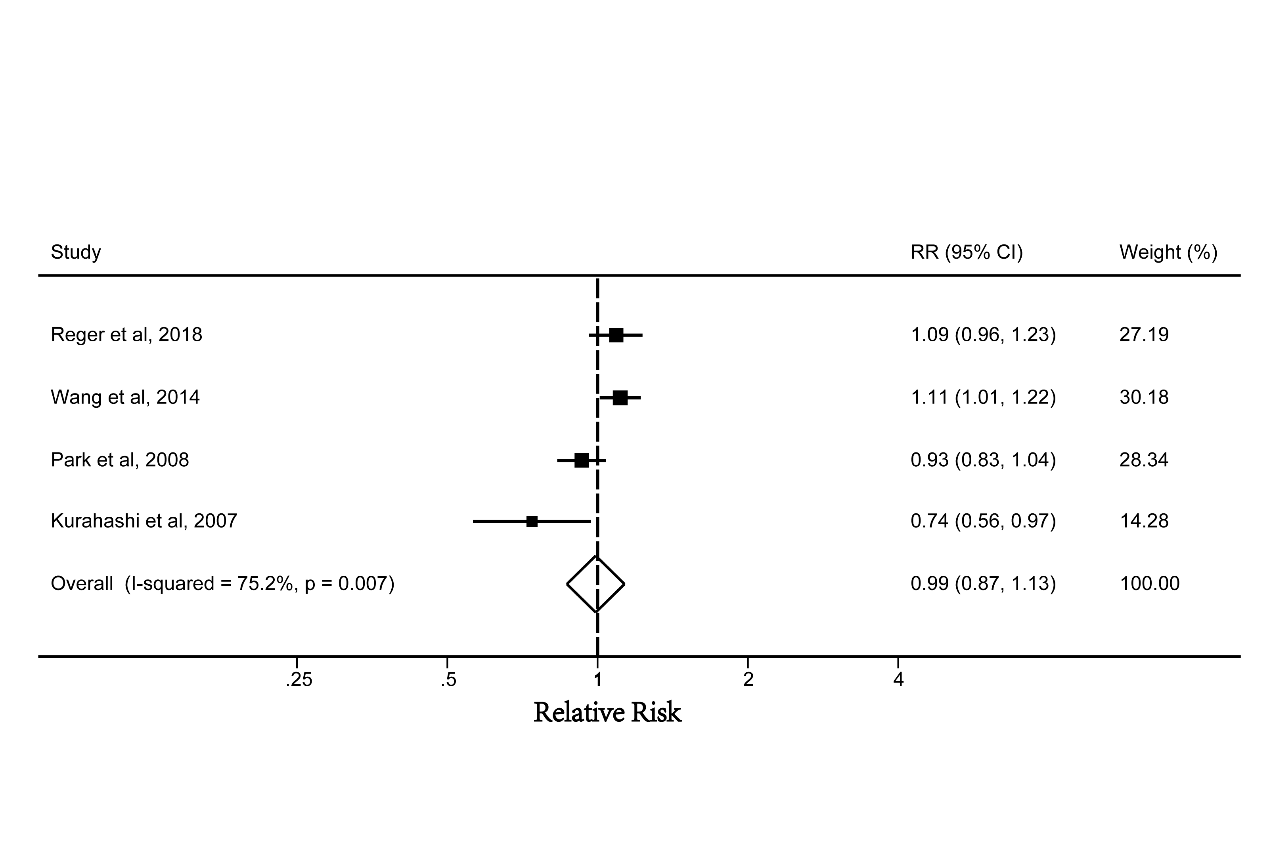
**

**Figure S13 ǀ** Forest plot for association between soy isoflavones intake and risk of prostate cancer, expressed as comparison between highest and lowest categories of soy isoflavones intake. The size of the black squares reflects the relative statistical weight of study-specific estimate, horizontal lines indicate 95% CIs. The diamond indicates the pooled RR estimates with 95% CI. CI, confidence interval; RR, relative risk.

**
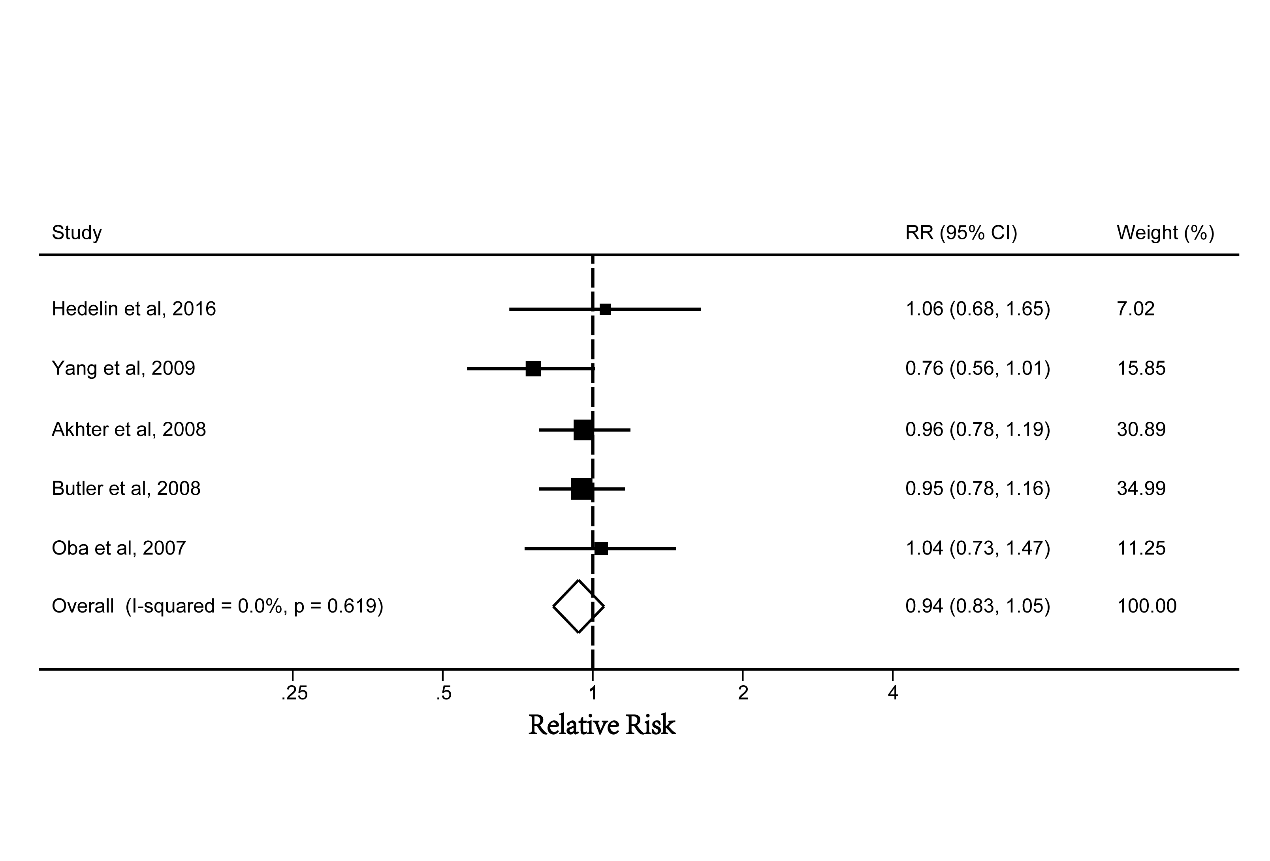
**

**Figure S14 ǀ** Forest plot for association between soy isoflavones intake and risk of colorectal cancer, expressed as comparison between highest and lowest categories of soy isoflavones intake. The size of the black squares reflects the relative statistical weight of study-specific estimate, horizontal lines indicate 95% CIs. The diamond indicates the pooled RR estimates with 95% CI. CI, confidence interval; RR, relative risk.

**
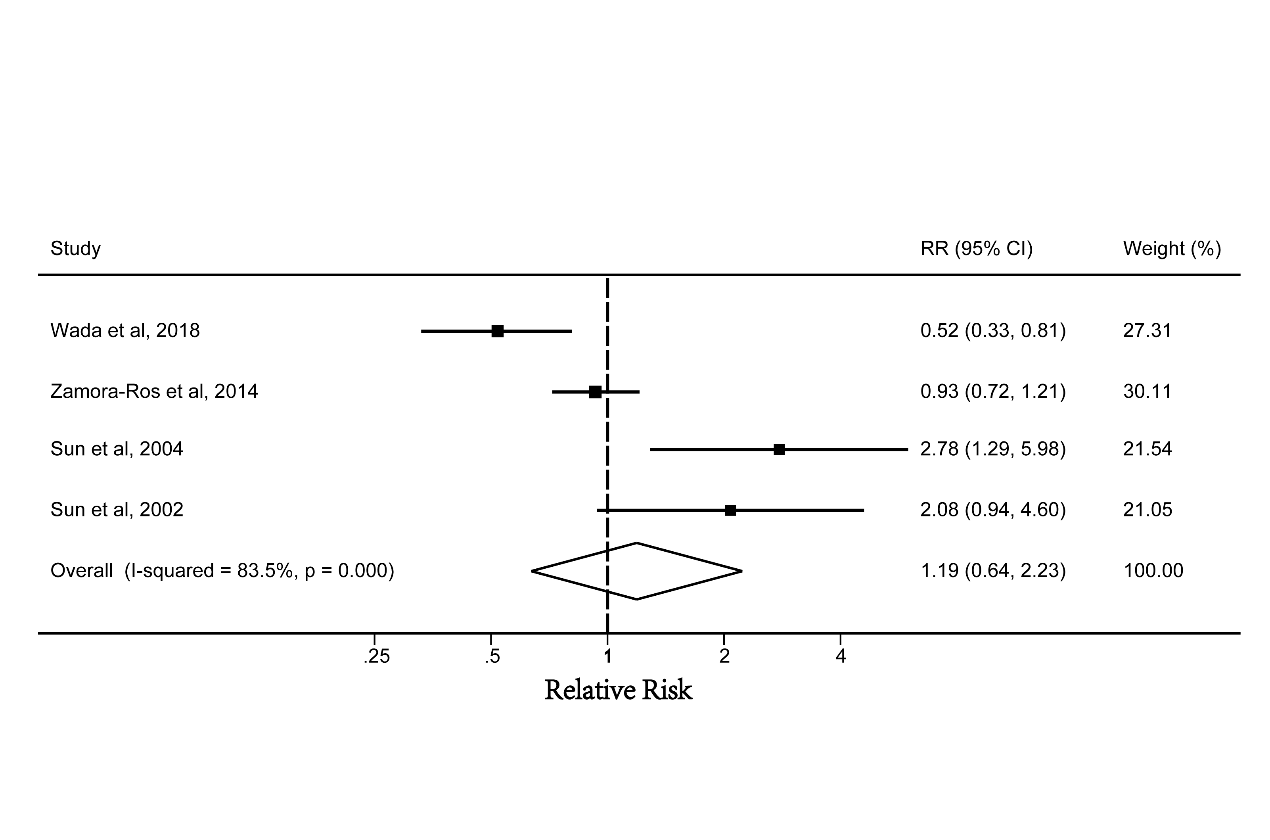
**

**Figure S15 ǀ** Forest plot for association between soy isoflavones intake and risk of bladder cancer, expressed as comparison between highest and lowest categories of soy isoflavones intake. The size of the black squares reflects the relative statistical weight of study-specific estimate, horizontal lines indicate 95% CIs. The diamond indicates the pooled RR estimates with 95% CI. CI, confidence interval; RR, relative risk.

**
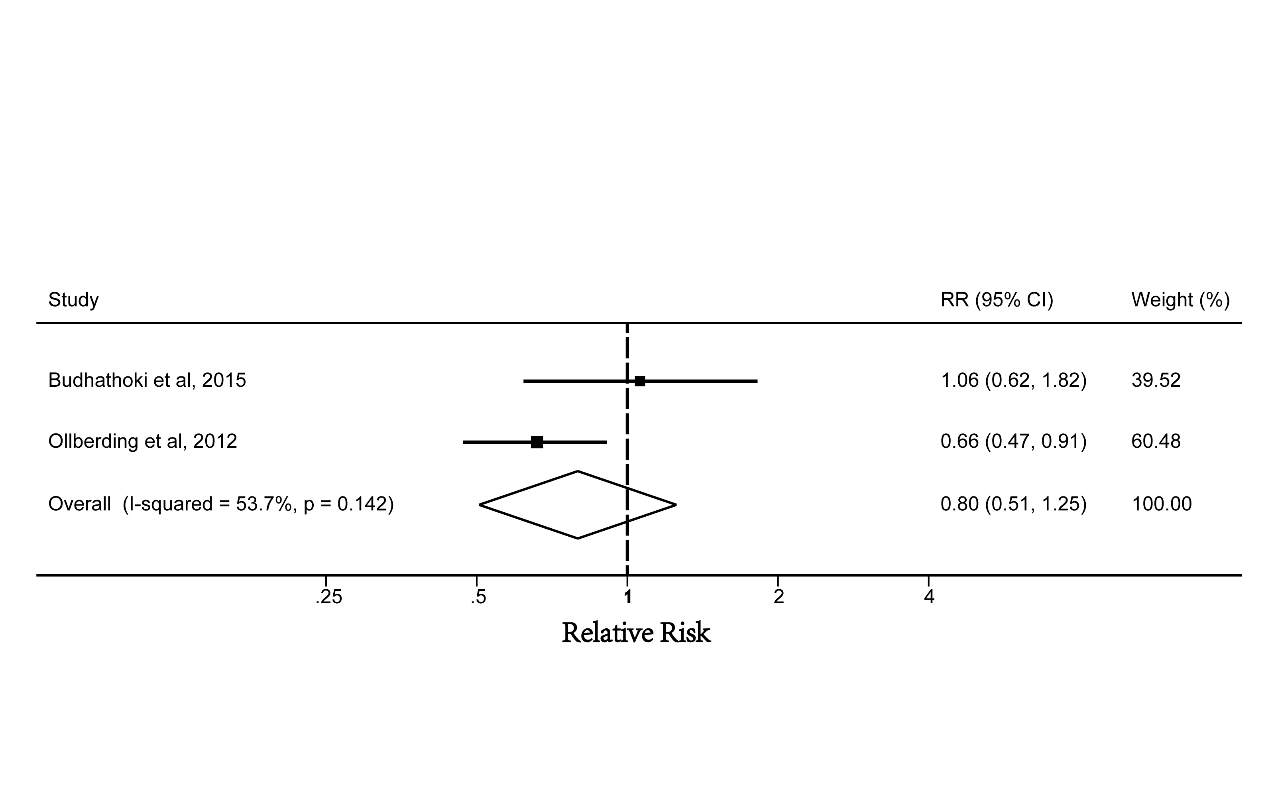
**

**Figure S16 ǀ** Forest plot for association between soy isoflavones intake and risk of endometrial cancer, expressed as comparison between highest and lowest categories of soy isoflavones intake. The size of the black squares reflects the relative statistical weight of study-specific estimate, horizontal lines indicate 95% CIs. The diamond indicates the pooled RR estimates with 95% CI. CI, confidence interval; RR, relative risk.

**
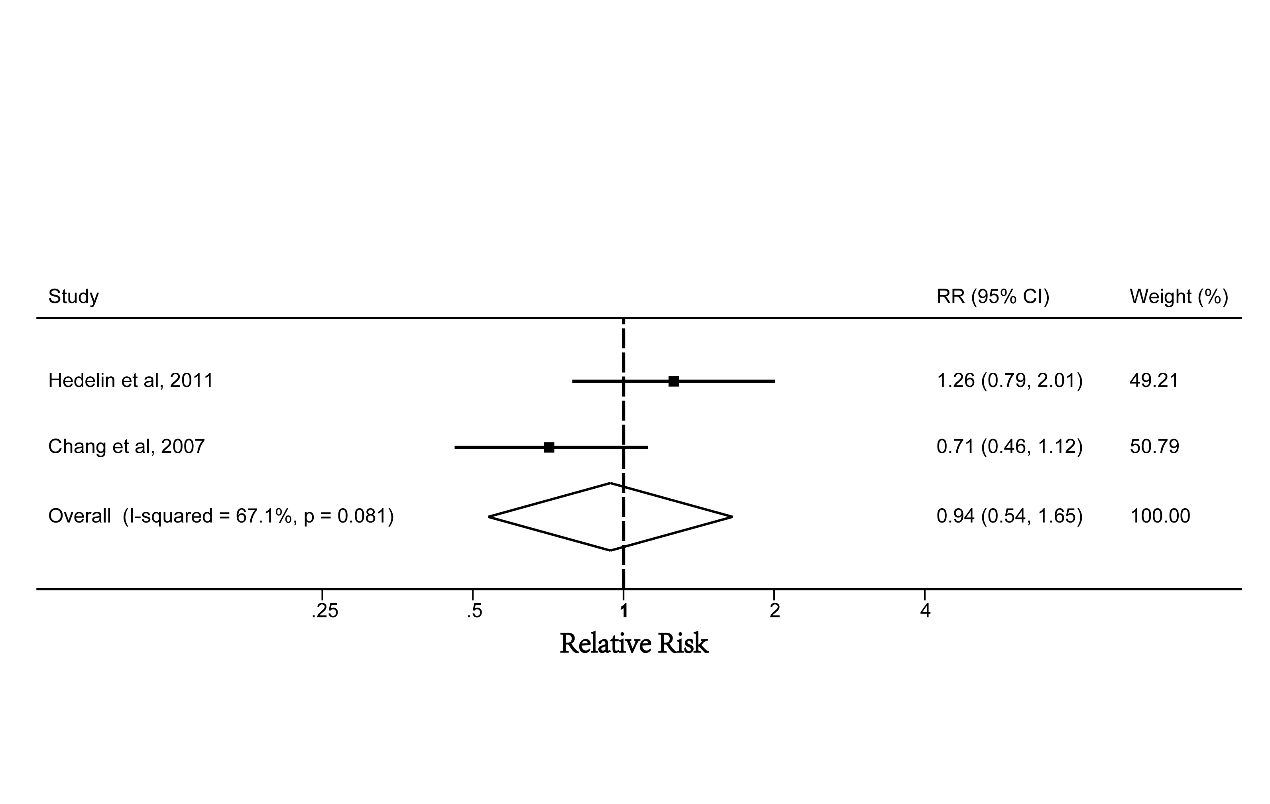
**

**Figure S17 ǀ** Forest plot for association between soy isoflavones intake and risk of ovarian cancer, expressed as comparison between highest and lowest categories of soy isoflavones intake. The size of the black squares reflects the relative statistical weight of study-specific estimate, horizontal lines indicate 95% CIs. The diamond indicates the pooled RR estimates with 95% CI. CI, confidence interval; RR, relative risk.

**
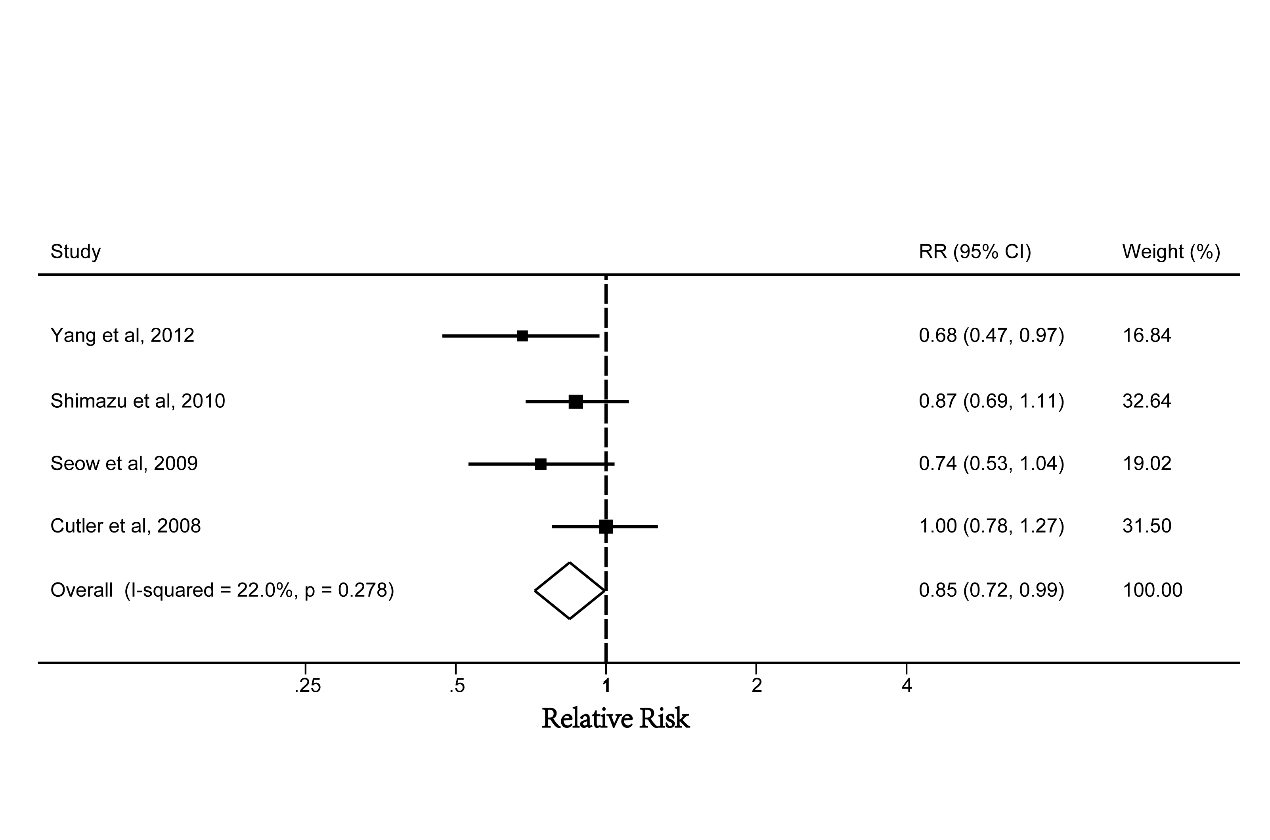
**

**Figure S18 ǀ** Forest plot for association between soy isoflavones intake and risk of lung cancer, expressed as comparison between highest and lowest categories of soy isoflavones intake. The size of the black squares reflects the relative statistical weight of study-specific estimate, horizontal lines indicate 95% CIs. The diamond indicates the pooled RR estimates with 95% CI. CI, confidence interval; RR, relative risk.

**
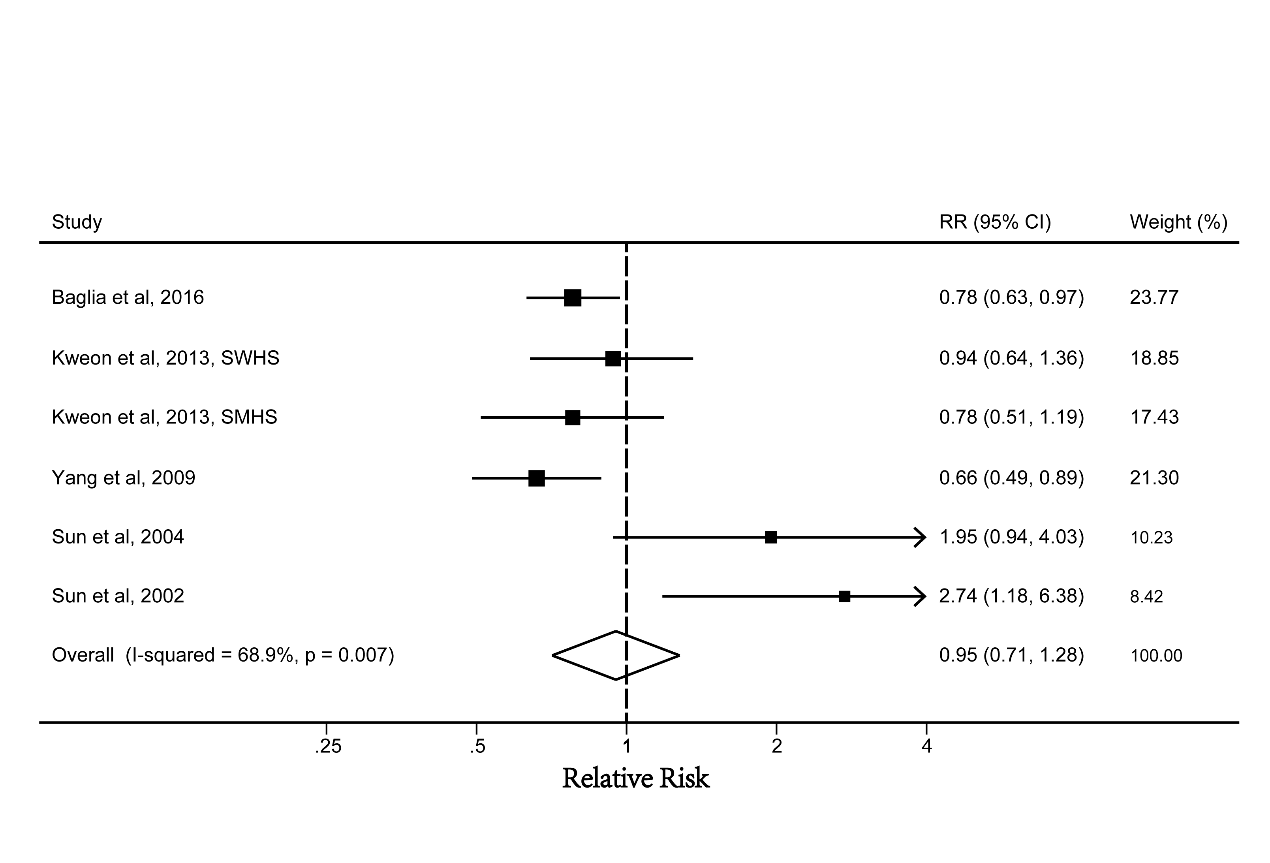
**

**Figure S19 ǀ** Forest plot for association between soy protein intake and risk of overall cancer incidence, expressed as comparison between highest and lowest categories of soy protein intake. The size of the black squares reflects the relative statistical weight of study-specific estimate, horizontal lines indicate 95% CIs. The diamond indicates the pooled RR estimates with 95% CI. CI, confidence interval; RR, relative risk; SWHS, Shanghai Women's Health Study; SMHS, Shanghai Men's Health Study.

**
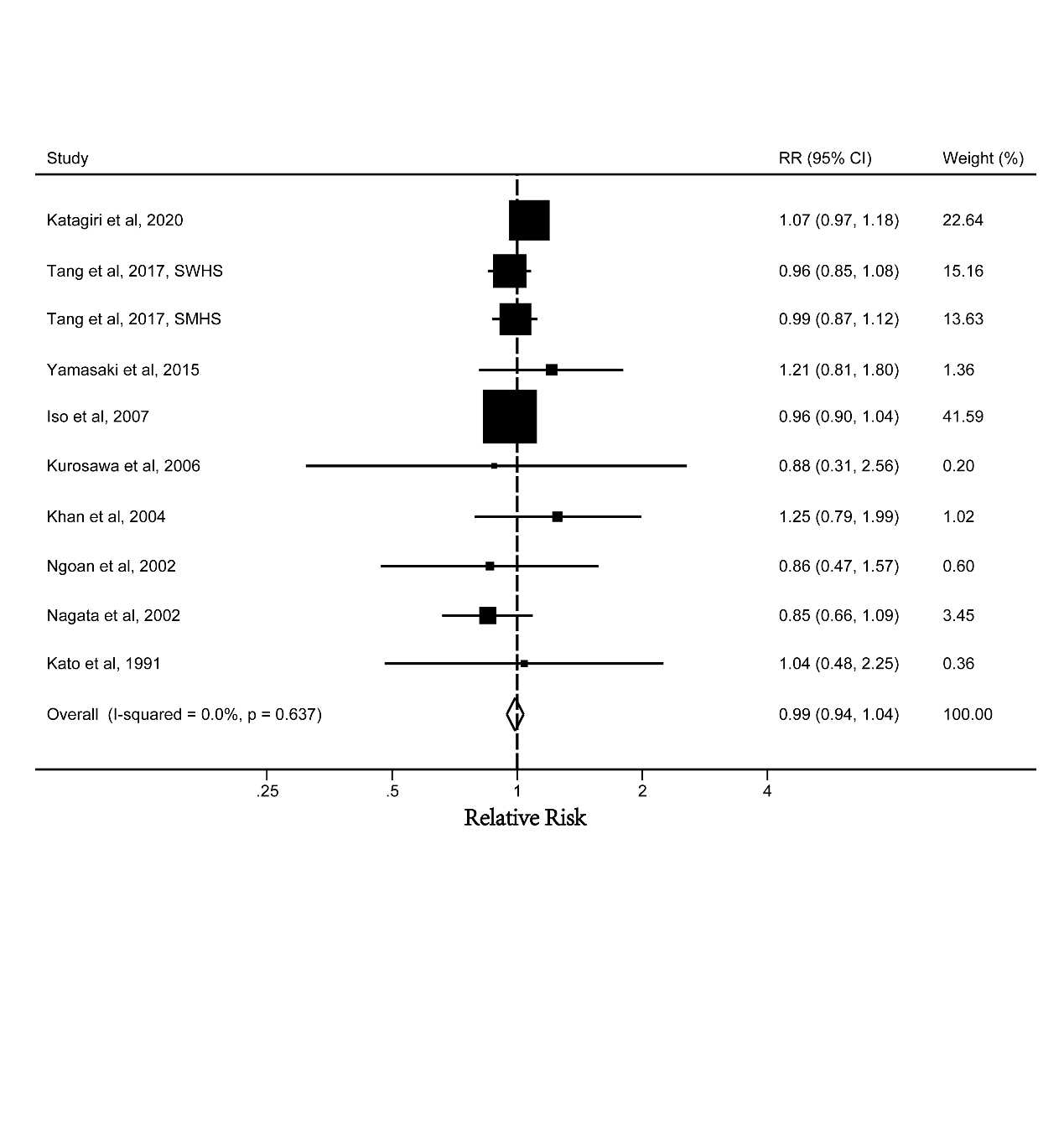
**

**Figure S20 ǀ** Forest plot for association between soy intake and risk of cancer mortality in general population, expressed as comparison between highest and lowest categories of soy intake. The size of the black squares reflects the relative statistical weight of study-specific estimate, horizontal lines indicate 95% CIs. The diamond indicates the pooled RR estimates with 95% CI. CI, confidence interval; RR, relative risk; SWHS, Shanghai Women's Health Study; SMHS, Shanghai Men's Health Study.

**
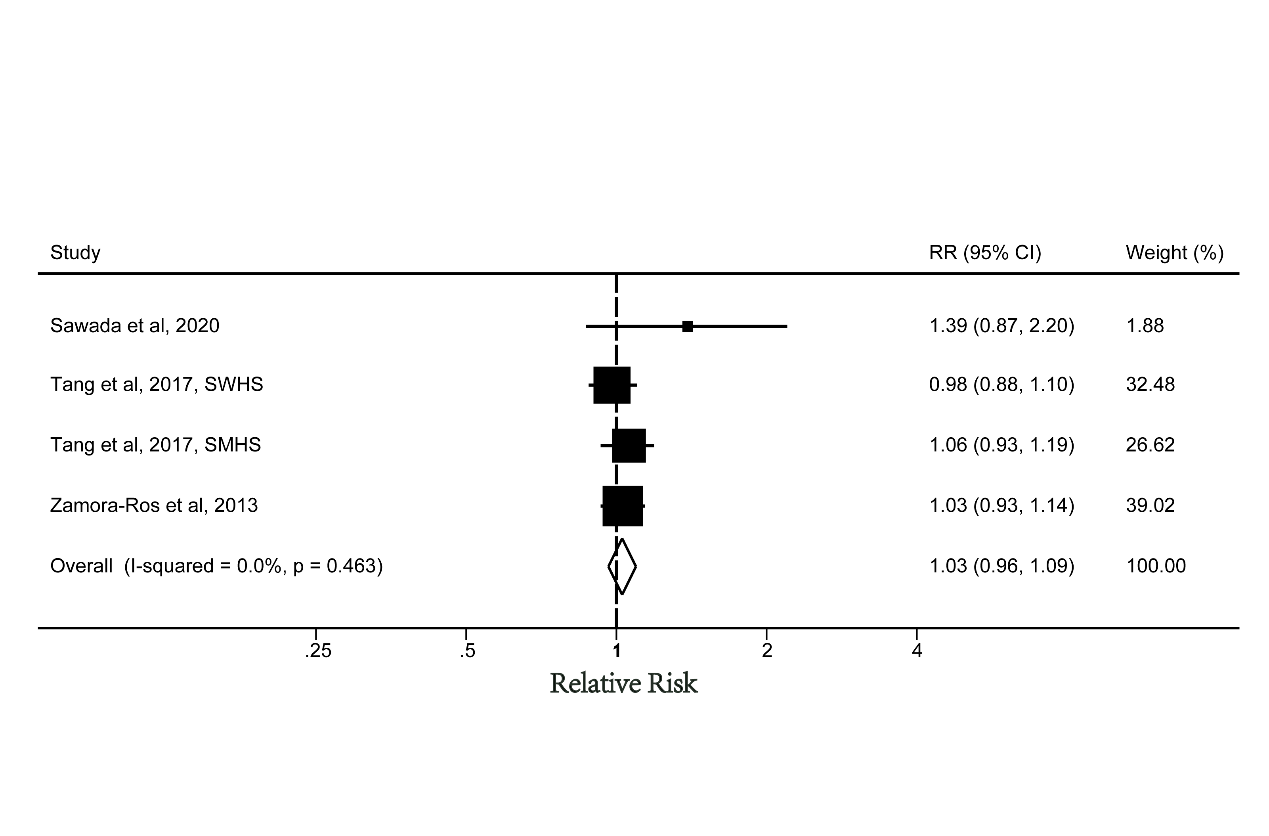
**

**Figure S21 ǀ** Forest plot for association between soy isoflavones intake and risk of cancer mortality in general population, expressed as comparison between highest and lowest categories of soy isoflavones intake. The size of the black squares reflects the relative statistical weight of study-specific estimate, horizontal lines indicate 95% CIs. The diamond indicates the pooled RR estimates with 95% CI. CI, confidence interval; RR, relative risk; SWHS, Shanghai Women's Health Study; SMHS, Shanghai Men's Health Study.

**
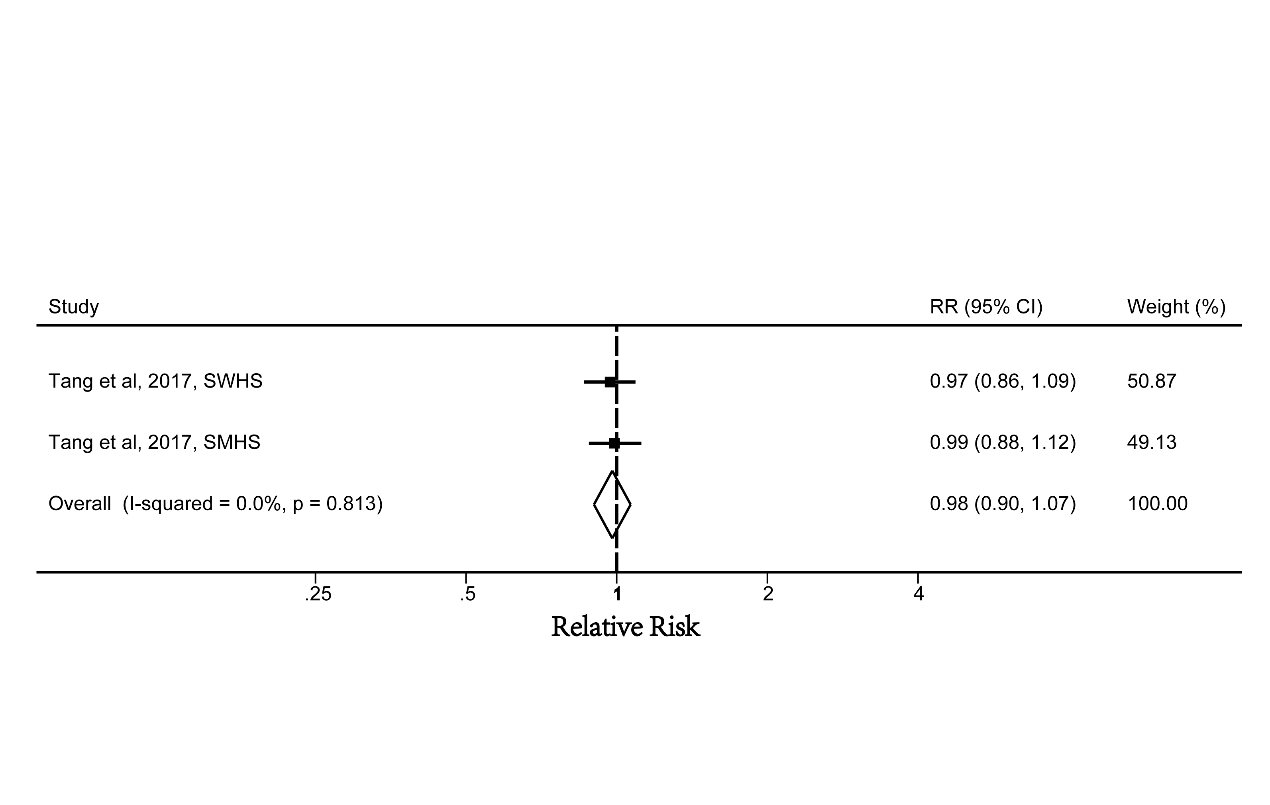
**

**Figure S22 ǀ** Forest plot for association between soy protein intake and risk of cancer mortality in general population, expressed as comparison between highest and lowest categories of soy protein intake. The size of the black squares reflects the relative statistical weight of study-specific estimate, horizontal lines indicate 95% CIs. The diamond indicates the pooled RR estimates with 95% CI. CI, confidence interval; RR, relative risk; SWHS, Shanghai Women's Health Study; SMHS, Shanghai Men's Health Study.

**
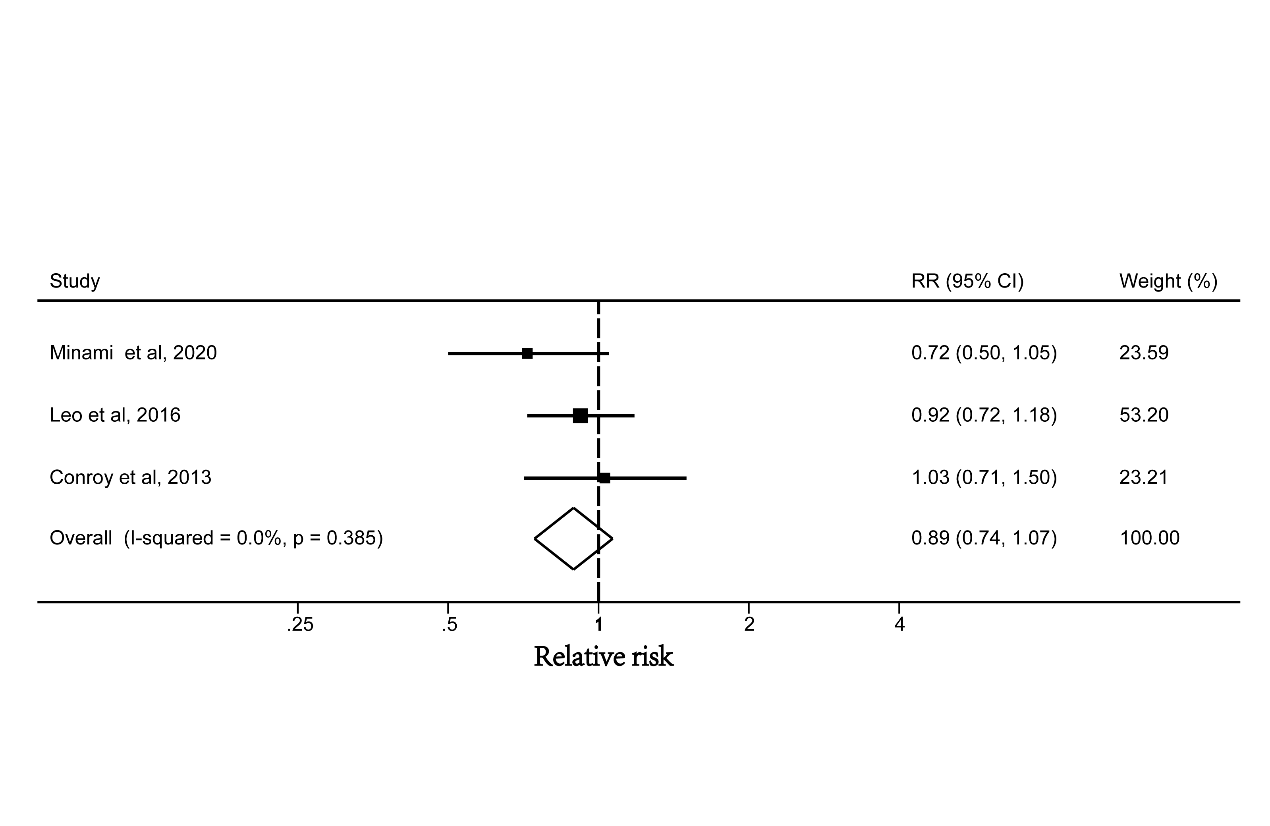
**

**Figure S23 ǀ** Forest plot for association between soy intake and risk of cancer mortality in cancer patients (i.e. cancer survival), expressed as comparison between highest and lowest categories of soy intake. The size of the black squares reflects the relative statistical weight of study-specific estimate, horizontal lines indicate 95% CIs. The diamond indicates the pooled RR estimates with 95% CI. CI, confidence interval; RR, relative risk.

**
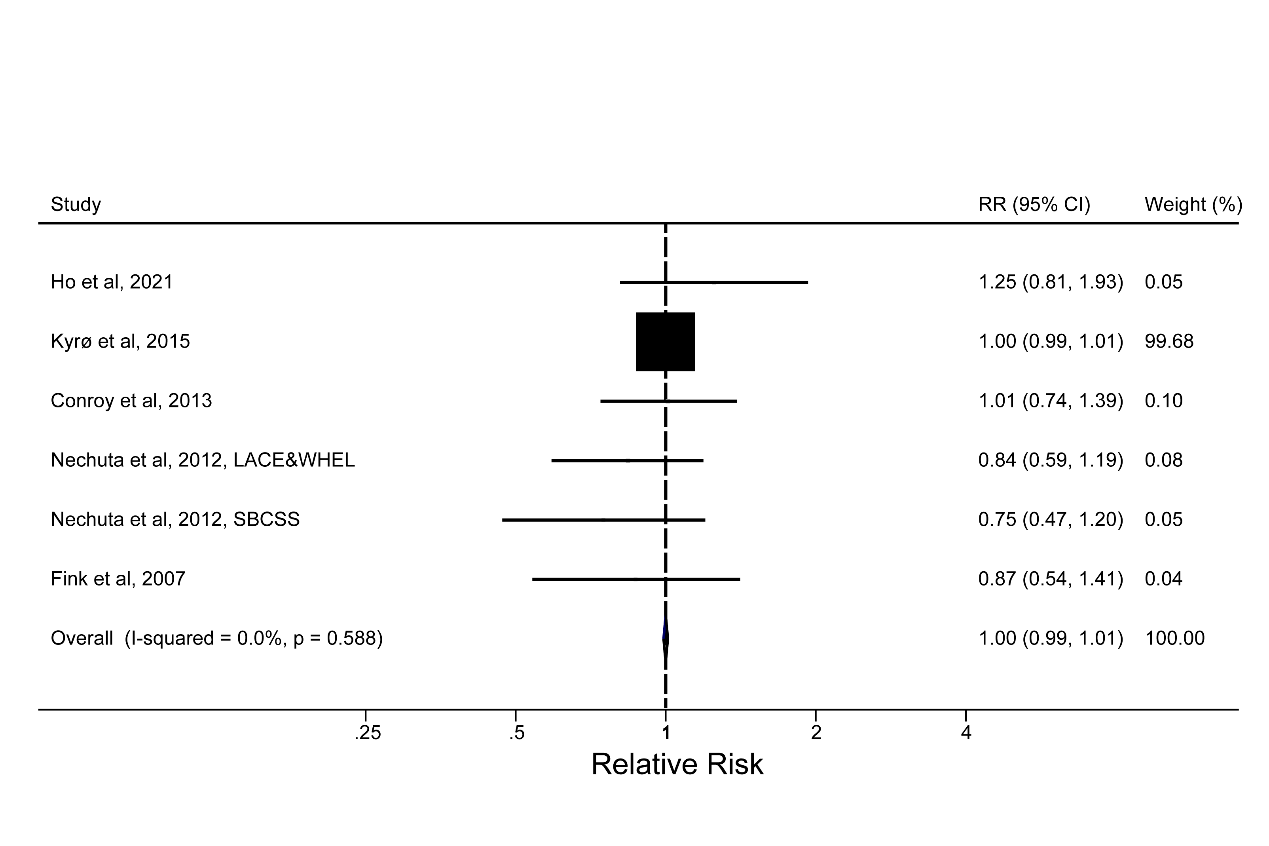
**

**Figure S24 ǀ** Forest plot for association between soy isoflavones intake and risk of cancer mortality in cancer patients (i.e. cancer survival), expressed as comparison between highest and lowest categories of soy isoflavones intake. The size of the black squares reflects the relative statistical weight of study-specific estimate, horizontal lines indicate 95% CIs. The diamond indicates the pooled RR estimates with 95% CI. CI, confidence interval; LACE, Life After Cancer Epidemiology; RR, relative risk; SBCSS, Shanghai Breast Cancer Survival Study; WHEL, Women’s Healthy Eating & Living.

**
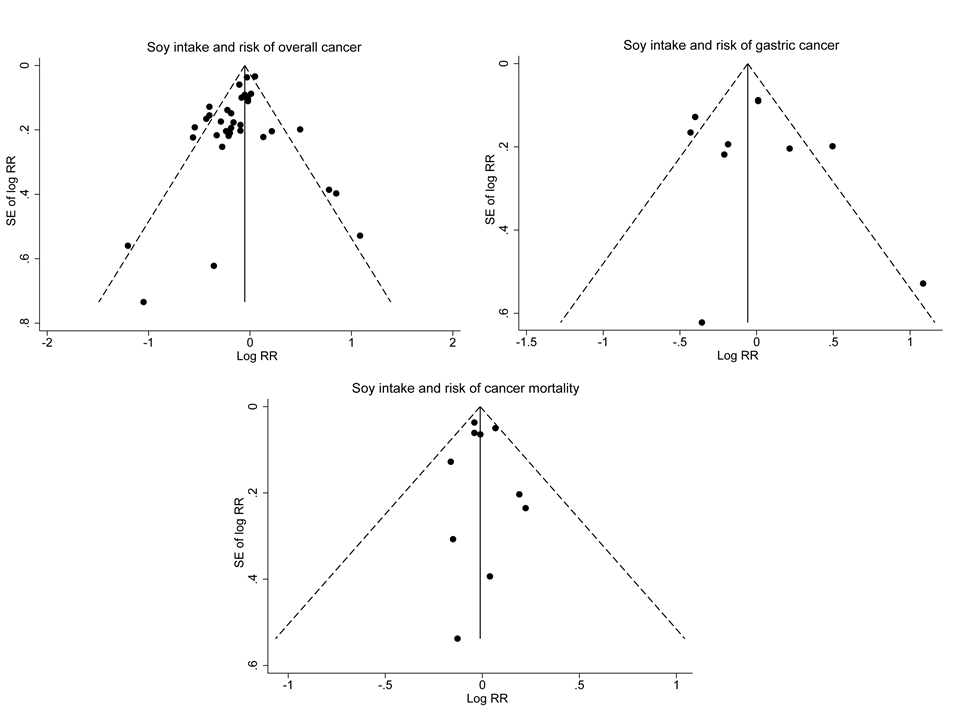
**

**Figure S25 ǀ** Funnel plot of association between soy intake and risk of cancer incidence and cancer mortality in general population. The vertical line represents the pooled RR. The dashed lines represent the pseudo-95% confidence interval of the RR. The circles represent risk estimates for each cohort. RR, relative risk. SE, standard error.

**
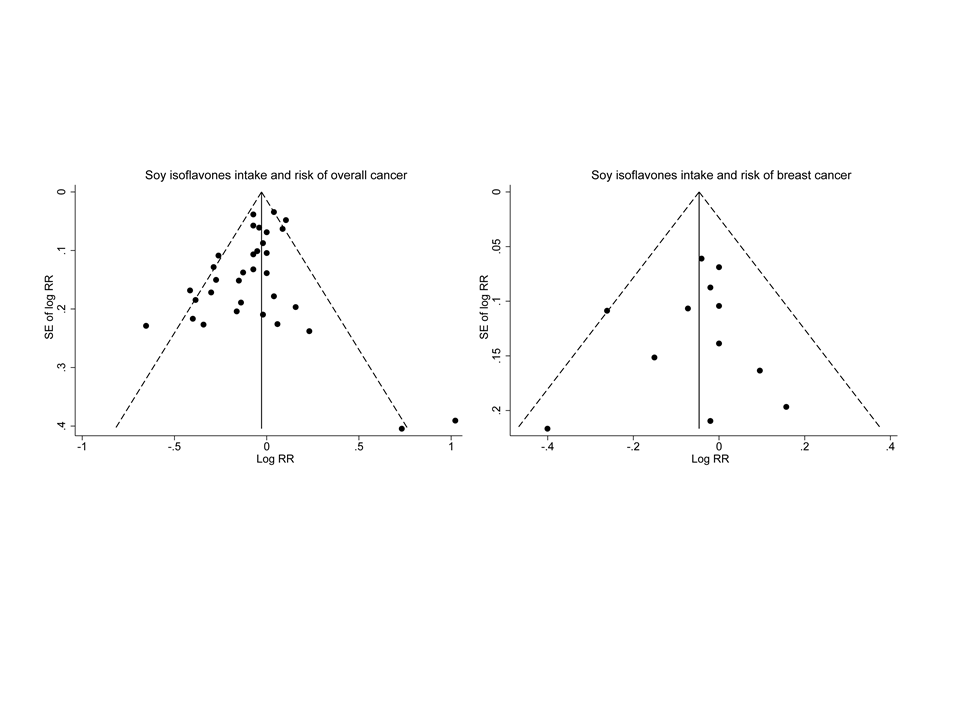
**

**Figure S26 ǀ** Funnel plot of association between soy isoflavones intake and risk of cancer incidence. The vertical line represents the pooled RR. The dashed lines represent the pseudo-95% confidence interval of the RR. The circles represent risk estimates for each cohort. RR, relative risk. SE, standard error.
